# The bacterial transcription terminator, Rho, functions as an RNA:DNA hybrid (RDH) helicase *in vivo*

**DOI:** 10.1042/BCJ20253089

**Published:** 2025-05-26

**Authors:** Ankita Bhosale, Ranjan Sen

**Affiliations:** 1 Laboratory of Transcription, Center for DNA Fingerprinting and Diagnostics, Uppal, Hyderabad 500039, India; 2 Graduate Studies, Manipal University, Manipal, Karnataka

**Keywords:** fluorescence microscopy, Rho, RNA:DNA hybrid, RNase H, *rut*-site, synthetic lethality

## Abstract

Ribonuclease HI (*rnhA*) removes the deleterious RNA:DNA hybrids (RDHs) by cleaving its RNA component. The bacterial transcription terminator Rho is an RNA-dependent 5′ → 3′ helicase capable of unwinding RDH formed on a single-stranded RNA *in vitro*. We hypothesize that Rho might be directly involved in RDH removal *in vivo*. Here, we demonstrate that Rho primary RNA-binding site (PBS) mutants defective in RNA binding and helicase activity are synthetically lethal specifically when RNase HI is absent. This lethality was not observed in the absence of RNase HII (*rnhB*) alone. Rho-PBS mutants in an *rnhA^-^* strain exhibited increased plasmid-concatemer and plasmid copy number, altered cell morphology, and were highly susceptible to DNA-damaging agents. These Rho mutants increased the accumulation of RDHs *in vivo*, suggesting defects in the RDH removal process. Rho was colocalized to RDHs *in vivo* when RNase HI was absent. Certain catalytically inactive mutants of RNase H that bind to the RDH blocked the entry of Rho to the RDH, inducing cell death, indicating the role of Rho in the removal of deleterious RDHs in the absence of RNase HI. Under *in vitro* conditions, Rho was capable of binding to the RDHs and unwinding them in a *rut*-site-dependent manner. Therefore, we concluded that in the absence of RNase HI, Rho, by its RNA-dependent helicase activity, is capable of unwinding RDHs in a *rut*-site-dependent manner. These results establish the non-transcription terminator role of Rho and its functional synergy with RNase HI *in vivo*.

## Introduction

In bacteria, the RNA:DNA hybrids (RDHs) form during transcription and replication processes [1,2]. RDHs are present in R-loops [3,4] and in Okazaki fragments [5]. During transcription, nascent RNA emerging from the elongation complex (EC) could form a duplex with the complementary DNA sequences in the melted regions of the template DNA. The Okazaki fragments are formed during the replication process upon extending the RNA primers on the lagging strands. These RDHs of the R-loops are primarily removed by the RNase H-mediated digestion of the RNA in these hybrids [1,6], whereas the RNA primers of the Okazaki fragments are primarily removed by DNA polymerase I, and RNase H plays an auxiliary role [7]. The accumulation of RDHs has a deleterious effect on cellular growth [8–12].

There are two types of RNase H in *Escherichia coli*: RNase HI and RNase HII. Both RNase Hs are distinct enzymatically. RNase HI cleaves the RNA component of any RDH that is ≥4 bp, whereas RNase HII cleaves at the RNA-DNA junction of the RDHs. Hence, the latter is also called junction ribonuclease [13–15]. RNase HI can remove the RDH (≥4 bp) of an R-loop [16]. Even though the deletion of these RNase Hs accumulates RDHs in the cell, they are not essential enzymes, suggesting the presence of other enzymes/factors that can remove the RDHs [17–19].

Transcription terminator Rho is a hexameric protein that has been involved in different physiological functions of the cell [20–31]. It has a primary RNA-binding site (PBS) at the N-terminus and a secondary binding site (SBS) at the C-terminus [32]. Rho binds specifically to the C-rich single-stranded *rut* sites (Rho-utilizations) through its PBS [33]. As RNA passes through the SBS, it activates the ATP-dependent RNA-helicase activities of Rho. The 5′ → 3′ helicase activity enables Rho to unwind RNA secondary structures (double-stranded RNA regions), and the RDHs [34] in the *in vitro* experiments provided that these structures are preceded by single-stranded RNA. These *in vitro* experiments revealed that Rho can exert its helicase function independently of its role as a transcription terminator.

It was earlier observed that the occurrence of R-loops [35–37], a structure that combines an RDH with a displaced DNA strand presumed to be formed during transcription elongation *in vivo*, was increased when Rho was inhibited with the Rho-inhibitor, bicyclomycin, in *E. coli* [38]. However, no functional relationship has been established between the Rho factor and RNase H activity for removing RNA from RDH and cellular survival. Since Rho can unwind RDHs formed on the mRNA in the *in vitro* experiments, we hypothesized that Rho could augment or complement the function of RNase H *in vivo* by directly removing the RDH structures.

Here, we systematically analyzed Rho’s role in RDH removal and complementation of RNase H function both *in vivo* and *in vitro*. A detailed analysis revealed that Rho PBS mutants specifically caused synthetic lethality when RNase HI was deleted but did not cause the same when RNase HII was deleted. Rho-PBS mutants induced the accumulation of RDHs *in vivo* and mimicked other physiological conditions observed when RNase HI was absent. Rho was seen to colocalize to RDHs *in vivo* when RNase HI was absent. Under *in vitro* conditions, Rho was capable of binding to the RDHs and unwinding them in a *rut*-site-dependent manner. Certain catalytically inactive mutants of RNase H that bind to the RDH also blocked the entry of Rho to the RDH mutants, leading to cell death, further indicating the importance of Rho in the removal of deleterious RDHs in the absence of RNase HI. These results provide new insights into the molecular mechanism by which Rho removes the RDHs and establishes its non-transcription terminator role and its functional synergy with the RNase HI *in vivo*.

## Results

### Hypothesis

*In vitro*, Rho is an aggressive RNA helicase capable of unwinding RDHs [34,35,39] and RNA secondary structures that it encounters along the RNA [27]. Rho executes this helicase function on any RNA stretches, independent of its termination function. Hence, it is expected that the Rho helicase function could unwind the deleterious RDHs and function as a ‘backup’ in the event of the failure of RNase H to function or when it is absent. RNase H is not essential to *E. coli*, indicating the existence of other mechanisms to remove the RDHs in the cell when the former is absent. We explored the role of Rho helicase in removing the deleterious RDHs *in vivo*.

### Synthetic growth defects of the Rho mutants in the absence of RNase HI

**Figure 1:**
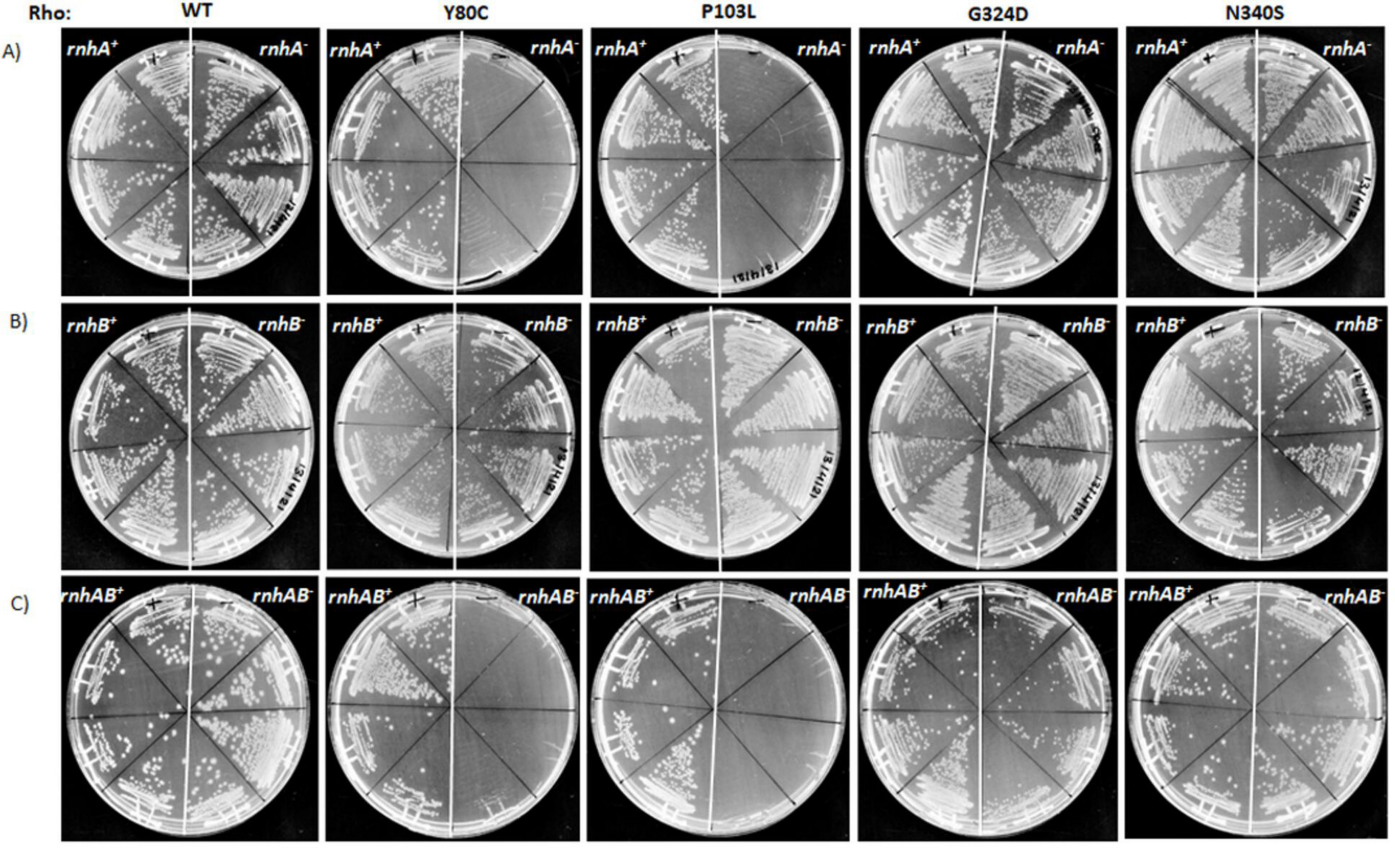
Synthetic lethality of RNase H with different Rho mutants. Growth on the LB plates of the Rho mutants under various conditions: In the absence of (**A**) RNase HI (*DrnhA*), (**B**) RNase H2 (*DrnhB*), and (**C**) both RNase HI and HII (*DrnhAB*). The left side of the panel shows ‘*rnhA*^+^, *rnhB*^+^ and *rnhAB*^+^’, indicating the presence of the indicated *rnh* on the chromosome, and the right side of the panel shows ‘*rnhA*^-^, *rnhB*^-^ and *rnhAB*^-^, indicating the deletion of the indicated *rnh*. After the deletion of the specific *rnh* and subsequently introducing the plasmids (pCl1920) expressing WT and the Rho mutants, the shelter Rho plasmid (IPTG-dependent pHYD1201 *amp*^R^) was removed by repeated streaking on LB-LB-spectinomycin plates without Ampicillin and IPTG. The final MG1655 strains were streaked on LB plates side by side, supplemented with spectinomycin.

Synthetic growth defect or lethality is defined as the simultaneous disruption of both genes, leading to lethality or a significant reduction in growth rate [40], a useful tool to establish the functional involvement of these two gene products. We chose four-point mutants (Y80C, P103L, G324D, and N340S) of Rho that were earlier characterized in our laboratory [41]. Among them, Y80C and P103L are in the Rho-PBS domain defective for RNA binding at the PBS and hence fail to activate ATPase and helicase activities in the presence of RNA. The G324D and N340S mutations are located in the C-terminal (Rho-SBS domain), and these mutants have weak ATPase activities with slow RNA helicase function but bind to RNA at their PBS normally. To establish the direct functional linkages between Rho and the RNase HI and HII, we tested for the lethality/growth defects caused by these Rho mutants [41] in the absence of either RNase HI (*DrnhA*) or RNase HII (*DrnhB*). Deletion of these *rnh* genes did not affect the growth of the cell in the wildtype (WT) MG1655 (Figure 1A-C, the WT Rho panels). In an earlier study, deletion of both the *rnhA* and *rnhB* induced slower growth [18]. We have also observed poor growth of *DrnhAB* in the MC4100 strain (Supplementary Figure S1C and D). Therefore, this inconsistency could be attributed to the genotypes of the different WT strains that are used in these two studies. We observed that the MG1655 strains expressing the Rho-PBS mutants did not grow when either *rnhA* or both *rnhA* and *rnhB* were deleted (Figure 1A and C). These Rho-PBS mutants grew normally in the presence of either WT *rho* or *rnhA* and *rnhB,* indicating that they don’t have any dominant effect and a synergy between WT *rho* and *rnhA* (Supplementary Figure S1). However, these mutants did not show any significant growth defects when only *rnhB* was deleted (Figure 1B). The Rho-SBS mutants did not show any growth defect when either or both of these two genes were deleted (Figure 1, the rightmost panels). Consistent with this observation, treatment of the *rnhA*, *rnhB,* or *rnhAB* deletion strains with the sub-lethal concentrations (20 mg/ml) of bicyclomycin (BCM) that bind at the Rho-SBS mutants impairs the helicase function [42] and also does not elicit synthetic growth defects (data not shown).

These results suggest that Rho specifically complements the function of RNase HI but not that of RNase HII, and its primary RNA-binding step is important for functional complementation. The PBS mutants do not bind to single-stranded RNA and also do not have helicase activity and hence, in the absence of RNase HI, might not be capable of unwinding the deleterious longer RDHs that the RNase HI usually removes. The SBS mutants have slow/weak helicase activities [41], and so does the BCM-bound Rho [42]. The slow helicase activities, as well as the slow removal of RDHs, were likely enough to support cell growth when RNase HI was absent. RNase HII removes the ribonucleotides that are incorporated in the DNA by mistake during replication [43], and the Rho helicase function might not be useful to perform this function of RNase HII.

### Physiology of Rho-PBS mutant mimics the *ΔrnhA* phenotypes

RNase HI is involved in DNA repair [11,44–47] and ColE1 plasmid replication [48–50] processes. Hence, the RNase HI deletion strains (*DrnhA*) exhibit sensitivity to UV and the DNA-damaging agent Mitomycin C [19] and increased ColE1 plasmid copy number, leading to higher concatemer formations [51,52]. If Rho performs the functions of RNase HI, it is expected that the Rho Y80C mutant in the absence of RNase HI would manifest synthetic defects in DNA repair and plasmid copy number control. Since the Rho Y80C mutant exerted severe growth defects in the MG1655 strain in the absence of RNase HI (Figure 1), we chose an MC4100 strain (RS257 and its derivatives, see Supplementary Table S1), in which the *rnhA rho Y80C* double mutants exhibited milder growth defects to perform the *in vivo* experiments (Supplementary Figure S1).

**Figure 2:**
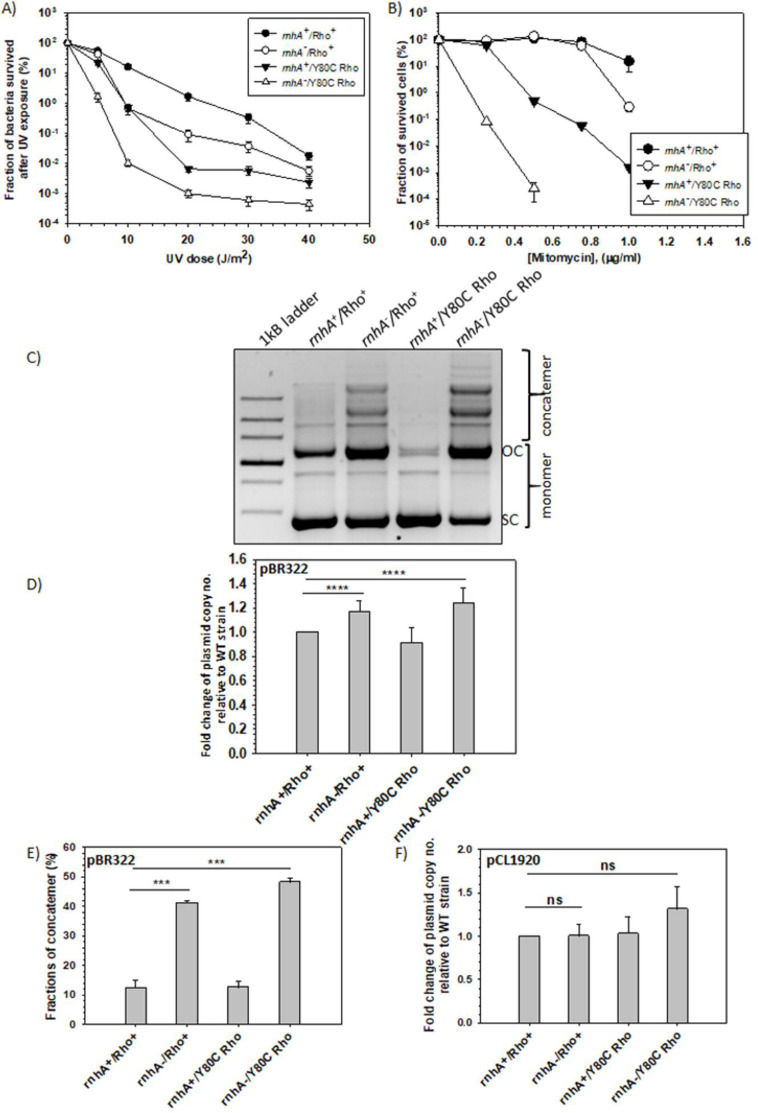
Physiology of the WT and the Y80C Rho mutant in the presence and absence of RNase HI. Plots showing the effects of different UV doses (J/m^2^) (**A**) and Mitomycin C doses (µg/ml) (**B**) on the survival of the indicated strains show the fraction of bacteria that survived after treatments. The fractions of surviving bacteria in both cases were calculated by the formula: [(Number of colonies from the plates after UV treatment or in the presence of Mitomycin)/(Total number of colonies from corresponding untreated plates)] × 100. The standard error of the mean (SEM) was calculated from three independent experiments. (**C**) Agarose gel showing the concatemer formation of the colE1 plasmid pBR322 in the indicated strains. Plasmids were isolated from the mid-log phase (~0.4OD_600nm_) cultures. An equal volume of plasmid isolated from each strain was loaded onto the agarose gel. (**D**) The plot shows the fold changes in plasmid concentration isolated from the indicated strains relative to the WT strain (*rnhA*^+^/Rho^+^) from the total intensities of all the DNA bands as shown in (**C**). The fraction of concatemer (**E**) of the plasmid isolated from the indicated strains was calculated from the band intensities of the gel shown in (**C**). (**F**) The plot shows the fold changes in non-ColE1 plasmid pCL1920 concentration isolated from the indicated strains relative to the WT strain (*rnhA*^+^/Rho^+^) isolated from the mid-log phase culture. The plasmid concentrations were calculated from the DNA band intensities in a similar way to that performed for the pBR322 plasmid.

UV dose (Figure 2A) and DNA-damaging agent Mitomycin C (Figure 2B) concentration-dependent sensitivity of bacterial growth assays are measures of recovery from the DNA damage by these agents. Deletion of RNase HI induces milder growth defects in the MC4100 strain upon these treatments (Figure 2A and B). In cases of the strains expressing Y80C Rho mutant when RNase H was deleted, the growth was highly affected even at lower doses/concentrations of these agents. The strain expressing Y80C Rho, even in the presence of RNase HI, was very sensitive to Mitomycin C (Figure 2B). These synthetic defects suggest that both Rho and RNase HI are part of the same DNA repair pathway, and Rho might complement the function of RNase HI when the latter is absent. It should be noted that the sensitivity of UV dose varies between different *E. coli* strains (Supplementary Figure S2).

RNase HI removes the RNA primer that initiates the DNA replication of the ColE1 plasmids, and its absence results in an increased concatemer formation that appears as multimers in the agarose gel [51]. We isolated the pBR322 plasmid, having a ColE1 replication origin, from the mid-log-phase cultures of MC4100 strains expressing WT or Y80C Rho in the presence or absence of RNase HI and analyzed the multimerization of the plasmids from the agarose gel (Figure 2C). We observed the following: (1) the total copy number of the plasmid increased in RNase HI deleted strains, and the increase is more in Rho Y80C/*rnhA^-^* (Figure 2D); (2) the fraction of concatemers/multimers in the gel increased significantly when Rho Y80C mutant was expressed in the absence of RNase HI (Figure 2E). The plasmid copy number did not change significantly when the same experiments were repeated with a non-ColE1 plasmid, pCL1920, that does not depend on RNase HI for its replication (Figure 2F). These results further support the involvement of Rho in the RNase HI functional pathway(s).

We also observed that due to enhanced DNA repair and replication defects in RNase HI functions in the absence of WT Rho, the MC4100 has a slow cell division process manifested as elongated and filamentous cell morphology with long DAPI-stained nucleoids under super-resolution microscopes (Supplementary Figure S3A).

### Enhanced accumulation of RDHs in the RhoY80C/rnhA^-^ strains

**Figure 3:**
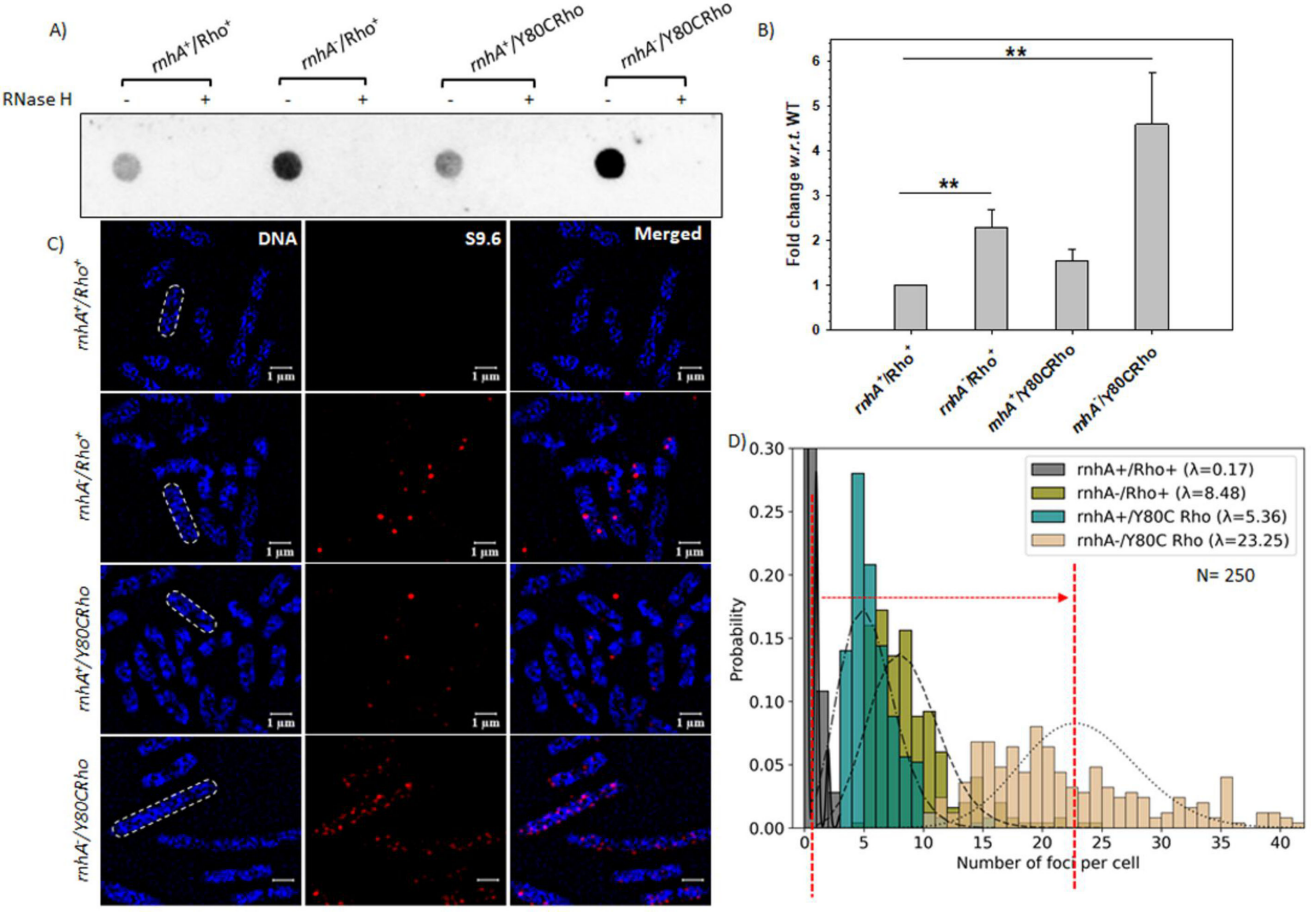
Accumulation of RDHs in the various strains. (**A**) Immunoblot of total nucleic acid preparations from log phase culture of the indicated strains using the RDH-specific monoclonal antibody mS9.6. 1 mg of the total DNA was extracted from the log-phase cultures of MC4100 with the indicated genotypes and was loaded in each well of the BioRad Dot blot apparatus. ‘-’ represents untreated samples; ‘+’ represents samples treated with 0.01U of RNase H (Epicentre) before blotting. (**B**) Quantitative analysis of the signal intensities of each of the blots expressed as fold increase relative to the preparation obtained from the WT strain. The error bars were calculated from three independent replicates. ** indicated *P* value ≤ 0.01. (**C**) Super-resolution microscopy of the MC4100 with the indicated genotypes. Cells were treated as per the methods described in the method section, following which immunofluorescence (IF) was performed with a rhodamine-tagged (red) secondary antibody against the RDH-specific primary antibody mS9.6. The same cells were also simultaneously stained with DAPI (blue) to visualize the chromosome. A merged image of DAPI and rhodamine in each case is also shown to identify the colocalized spots of the rhodamine-stained RDH and DAPI-stained chromosome. The scale bars indicated are 1 mm. (**D**) The Poisson distribution plot shows the number of foci per cell in the case of *rnhA*^+^/Rho^+^ (gray), *rnhA^-^*/Rho^+^ (olive), *rnhA*^+^/Y80C Rho (teal), and *rnhA*^-^/Y80C Rho (Wheat). The plot was generated by a dedicated Python-inbuilt script. λ, the Poisson distribution rate, represented the average of the foci per cell. *N* is the total number of cells used for distribution analyses. The red dashed line represents the average value in the cases of *rnhA*^+^/Rho^+^ (left) and *rnhA*^+^/Y80C Rho (right). The arrow indicated the change in the Poisson distribution rate. RDH, RNA:DNA hybrid.

The above results reflected the consequences of the cell physiology when the deleterious RDH removal function is lacking and suggest enhanced accumulation of RDH structures in the MC4100 strains expressing Rho Y80C mutant in the absence of RNase HI. Hence, we measured the levels of RDH structures on the bacterial chromosome using the RDH-specific monoclonal antibody, S9.6, by immunoblotting and visualized these structures in a super-resolution microscope by immunofluorescence (IF) (Figure 3). Immunoblotting experiments described in Figure 3A and B show that the RDH level of the log-phase culture of the strain *rhnA*^-^/Y80C Rho significantly increased by ~4.5 fold relative to that observed in the WT. A moderate increase (1.5- to 2-fold) in the level of RDH was also observed in *rhnA*^+^/Rho Y80C and *rhnA*^-^/Rho^+^ strains. Upon RNase H treatment, the immunoblot signals disappeared (Figure 3A), confirming the presence of RDH structures that were specifically detected by the antibody. We also probed the RDH structure formation on the chromosome by performing IF experiments using a super-resolution microscope. The mid-log phase cultures were treated with the m9.6 mouse antibody in conjugation with a secondary rhodamine-tagged anti-mouse antibody (Figure 3C and D). To quantitatively analyze the number of RDH spots formed on the chromosome in each cell of different strains, we first determined the median size of the cells for each strain from the Gaussian distributions, as shown in Supplementary Figure S4A-D. Rho Y80C-expressing cells in the absence of *rnhA* were highly elongated (median length of ~6 μm) due to impaired cell division. It should be noted that these elongated cells have a single chromosome, except that they are more spread out compared with the smaller cells. Since the RDH spots can only form on the chromosome, their number in each of the strains will vary based on their genotypes and not due to the length of the cells. Panels of Figure 3C show the RDH foci on the bacterial chromosome in each strain.

**Figure 4:**
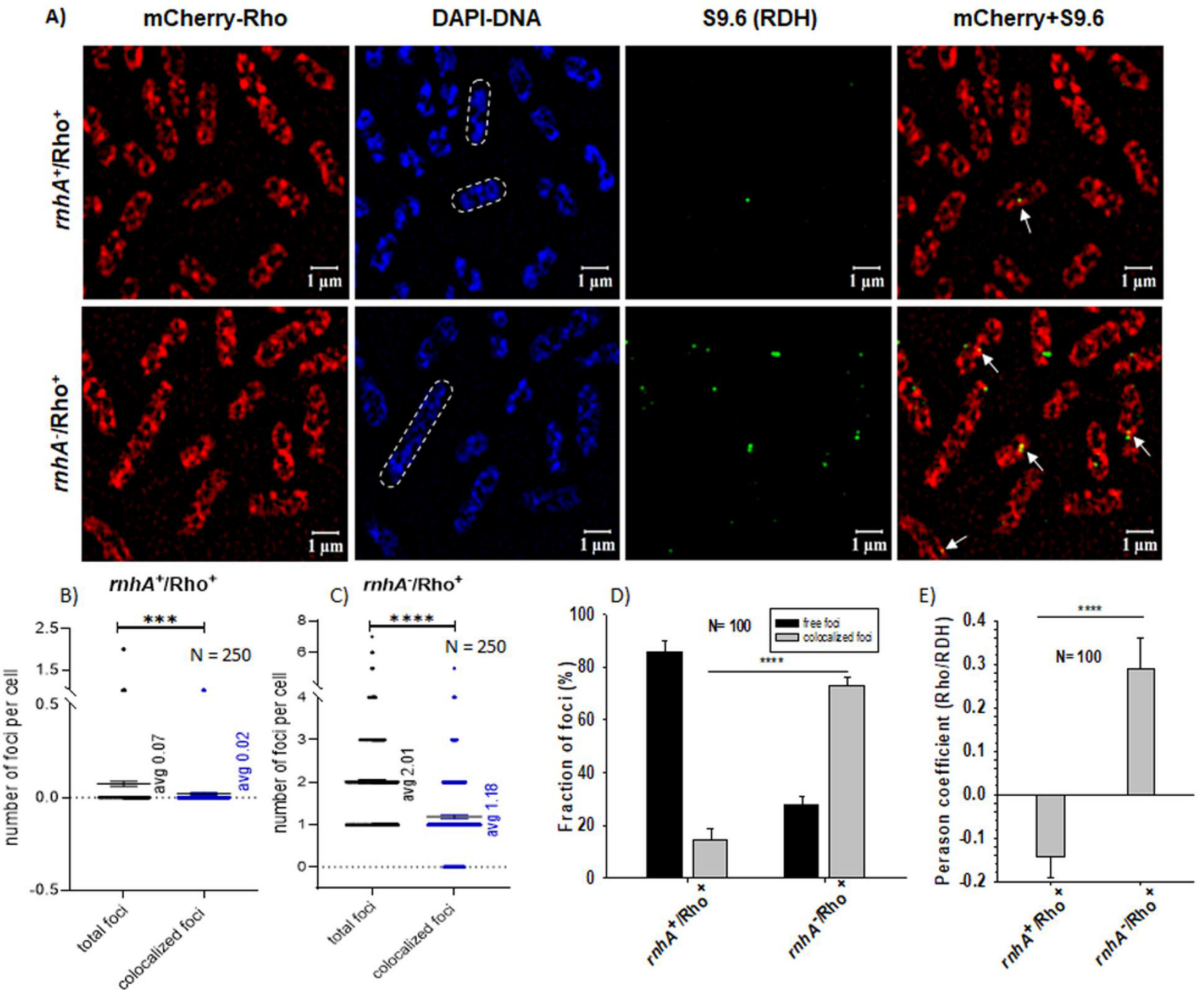
Colocalization of Rho-mCherry molecules on the RNA:DNA hybrids probed by immunofluorescence using mS9.6 mouse antibody. (**A**) Super-resolution microscopy of the *E. coli* MC4100 cells (*Rho^+^/rnhA^+^* and *Rho^+^/rnhA^-^*) expressing mCherry-WT Rho (red) was subjected to IF with the mS9.6 mouse antibody tagged to Alexa-fluor 488 (green) to probe the RDHs formed on the chromosome (blue). The right-most panels are the merged images of the red mCherry-Rho and the green RDHs. The red and green overlapped spots appeared as yellowish spots indicated by white arrowheads. The scale bar indicated by white lines is + mm. The total number of mS9.6 foci and colocalized mCherry-Rho-mS9.6 foci was counted from ≥200 cells by visual inspection. The scattered plots show the number of foci per cell in the case of *rnhA*^+^/Rho^+^ (**B**) and *rnhA*^-^/Rho^+^ (**C**). The t-test (unequal variance) was used for the determination of statistical significance. The error bar represents SEM. (**D**) The grouped column graphs show fractions of colocalization of the mCherry-Rho and S9.6 (RDH) spots in the indicated strains. *N* represents the number of cells used for analyses. The error bars represent the SEM. (**E**) Statistical significance of Rho colocalization with RDH estimated by Pearson correlation coefficient (PCC) analysis based on the scattered plots described in Supplementary Figure S6 using Zen Blue software. Error bars represent the SEM. *N* represents the number of cells used for analyses. *** indicated *P* value ≤0.001 and **** indicated *P* value ≤0.0001.

We employed the Poisson distribution statistics to analyze the average number of RDH foci per cell (rhodamine fluorescence spots localized on the DAPI-stained bacterial chromosome). The analyses revealed that the total foci in the *rhnA*^-^/Y80C Rho strain have increased significantly (the Poisson rate l rises from 0.17 to 23.25; Figure 3D). The same trend in the Poisson distribution curves was obtained when we normalized the number of foci per cell per median mm for each strain (Poisson rate l increases from 0.09 to 6.28 in *rhnA^-^/Y80C* strains compared with its WT counterpart, Supplementary Figure S4). Similar to the immunoblots (Figure 3A and B), the average number of RDH foci per cell in *rhnA*^-^/Rho^+^ and *rhnA*^+^/Y80C Rho was also increased to a lesser extent relative to the *rhnA*^+^/Rho^+^ strain (Figures 3D and 4E). These results suggested that cells with the Y80C Rho mutant in the absence of RNase HI were severely impaired in removing the RDHs. The WT Rho in the absence of RNase HI or the latter in the presence of the Rho mutant were partially capable of eliminating the RDHs. These data suggest Rho’s involvement in the elimination of RDHs when RNase H fails to function.

It should be noted that the treatment of the cells for IF experiments described here perturbs their integrity compared with when they were only paraformaldehyde-fixed before the imaging (compare Figure 3A and B). Cells appeared to be perforated, and the chromosomes had a fragmented appearance after mild cell lysis treatments to make the antibodies permeable to the cells. However, the RDH spots were always observed on the chromosome fragments.

### Colocalization of Rho onto the RDH structures

If Rho can remove the RDHs, it is expected that Rho should be visualized in the vicinity of these RDH structures. To visualize Rho in the cells, we transformed the WT or *rnhA^-^* MC4100 strain with a pCL1920 plasmid expressing an N-terminal-mCherry-tagged Rho [53]. In these strains, subsequently, the chromosomal Rho was deleted. Since the Rho-mediated RDH removal process would be transient, to trap these short-lived Rho-RDH species, we treated the cells with a short 30” pulse of formaldehyde to induce the *in vivo* crosslinking of Rho to RDHs. The mCherry-Rho expressing cells (red) were subjected to IF using an mS9.6 antibody and were visualized by super-resolution microscopy. Supplementary Figure S5A shows the natural distribution of mCherry-Rho along the membrane and at the poles in a mid-log phase culture that is not subjected to formaldehyde crosslinking treatment. After the IF protocols (Supplementary Figure S5), Rho remained on the membrane, but as the membrane was punctured, the continuous distribution of Rho was also affected. When there was formaldehyde treatment, Rho appeared to form foci at different parts of the cells, including on the DAPI-stained DNA (Supplementary Figure S5) in both cases when either the IF protocols were followed or not followed (Supplementary Figure S5). The DAPI-stained DNA configurations also changed upon formaldehyde treatment and after employing the IF protocols.

We visualized both the mCherry-Rho and the RDH spots (probed by mS9.6 antibody) on the bacterial chromosome (DAPI-stained) of the above-described cells. To visualize the colocalization of the red mCherry to the green spots (an Alexa Fluor 488-labeled secondary antibody of the mS9.6) of RDHs on the DAPI-stained (blue) chromosome, we merge the red mCherry and the green mS9.6 IF images to obtain the yellowish colocalized spots (Figure 4A). From a visual inspection of ~250 cells, in the absence of RNase HI (*rnhA^-^*), on average there were 2 RDH (green spots) foci per cell, and out of those, on average 1.2 foci overlapped (or colocalized) with the red mCherry foci, giving rise to yellowish spots (Figure 4C). The number of such colocalized spots was significantly less when mCherry-Rho was expressed in the presence of WT RNase HI (Figure 4B). To further validate these visual observations, we quantitatively analyzed the fraction of colocalized spots using the Zen 3 software built into the microscope. The bar graphs (Figure 4D) showed that the fraction of colocalized foci of Rho with RDH was increased from 14% to 72% when *rnhA* was deleted, following similar trends of the visual inspections. To further estimate the statistical significance of the occurrence of the Rho and RDH colocalized spots in the absence of RNase H in the cells, we performed the Pearson correlation coefficient (PCC) analysis of the green (RDH signal) and red (Rho signal) signal intensities in a cell. To obtain the PCC values, we constructed scattered plots of the intensities of the green and the red spots and estimated the colocalized spot intensities from the third quadrant of these plots (Supplementary Figure S6). We obtained the PCC value of the colocalized spots in the *rnhA*^-^/Rho^+^ strain as 0.221, indicating a positive correlation, whereas it was -0.142 in the *rnhA*^+^/Rho^+^ strain, indicating no or negative correlation between the occurrence of the signals emanating from mCherry-Rho and S9.6-labeled RDHs. We concluded that the occurrence of the colocalized spots is statistically significant. These results suggested that a significantly high number of Rho molecules stay in the vicinity of the RDH engaged in removing the latter structures when RNase HI is absent from the cells.

### The D10NrnhA or D70NrnhA mutants of RNase H prevent Rho from removing the RDH structures *in vivo*

**Figure 5:**
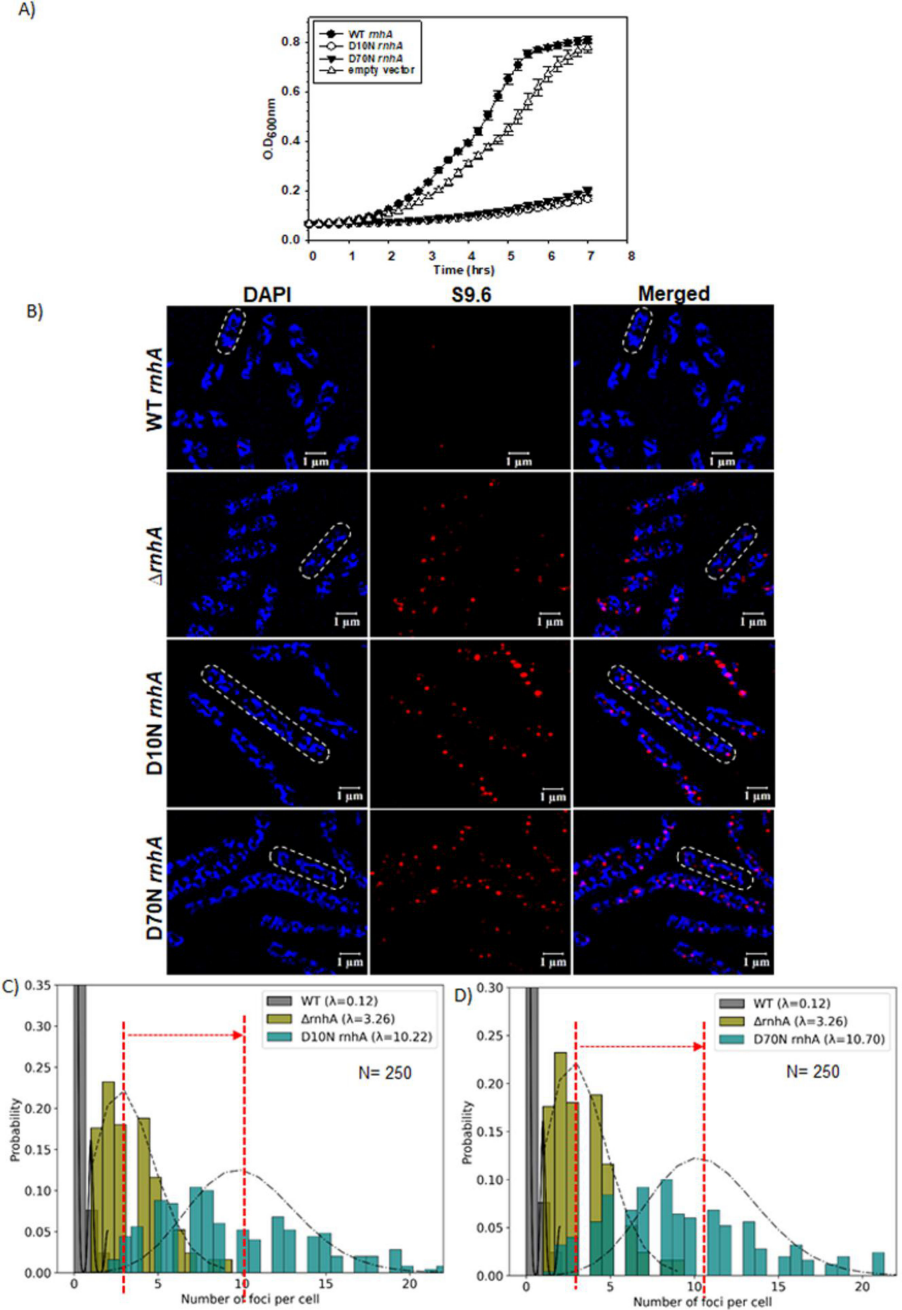
Effect of rnhA point mutations on cell growth and RNA:DNA hybrid formation. (**A**) Growth curves showing the varying degrees of growth defects upon deletion of the *rnhA* and in the presence of *rnhA* point mutants, D10N and D70N, and growths were compared with the WT strain (*rnhA*^+^). In all these strains, the chromosomal *rnhA* was deleted, and the indicated *rnhA* alleles were introduced in the cells from the low-copy-number vector (pOK12). The standard error bars were obtained from three independent measurements. (**B**) Super-resolution microscopy showing the probing of the RDHs with mS9.6 antibody and corresponding rhodamine-tagged secondary antibody of the indicated strains (red puncta). The DNA was stained with DAPI (blue). The merged images indicate the number of red puncta representing the RDHs on the DAPI-stained DNA. The scale bar indicated as white lines is 1 µm. (**C**) The Poisson distribution plots show the number of foci per cell in the case of WT (gray), ∆*rnhA* (olive), and D10N *rnhA* (teal). (**D**) D70N *rnhA* (Teal). The plots were generated by Python’s built-in script. λ, the Poisson distribution rate, represented the average of the foci per cell. *N* is the total number of cells used for distribution analyses. RDH, RNA:DNA hybrid.

Rho should require direct access to RDHs to remove them *in vivo*. Hence, if RDH structures are tightly bound to RDH-binding proteins *in vivo*, Rho would not be able to access them and remove them, which would also be a piece of evidence that it is directly involved in removing these structures *in vivo*. Previously, it was shown that the point mutants D10N and D70N of RNase HI bind strongly to RDH but are incapable of cleaving their RNA components *in vitro* [54–56]. However, it is not known whether they exhibit similar activities *in vivo*. We constructed these two mutants along with the WT *rnhA* in a low-copy pOK12 vector [57] for the *in vivo* assays (Supplementary Table S2). Since RNase H is not essential, *E. coli* MC4100 (RS257) grew normally in its absence (Supplementary Figure S1). However, the expression of these mutants in the absence of the WT RNase H caused severe growth defects (Figure 5A). This severe growth defect was also reflected in the highly elongated cell morphology of these mutants (Supplementary Figure S7A), which suggests impairment of cell division. To calculate the average cell length and the number of RDH spots in these cells, we employed a Gaussian distribution by measuring the length of ~500 cells, as shown in Supplementary Figure S8. The cell length analyses revealed that the D10N and D70N mutants are ~8–9 fold longer than the WT. Similarly, as described in Figure 3, the RDH foci were probed in these mutant cells by mS9.6 antibody (Figure 5B), revealing a significantly high number of RDH foci in the RNase H mutants compared to the ∆*rnhA*. It should be noted that even though the D10N and D70N cells are very elongated, they have a single chromosome on which the RDHs can form. Hence, the increase in RDH spots will only be due to impaired removal of these structures in the mutants and will not be dependent on the length of the cells. We employed Poisson distribution statistics to analyze the average number of RDH foci per cell (rhodamine fluorescence located on the DAPI-stained bacterial chromosome). The analyses revealed that the total foci in the RNase H mutants have increased to a significantly high value (Poisson rate l rises from 0.12 to ~10; Figure 5C and D). The increase in the number of spots increased moderately in the strains where RNase H was deleted (Poisson rate l rises to ~3.26).

Enhanced spot intensities in these two RNase H mutant-expressing cells in the immunoblotting experiments also supported the conclusion of the presence of a high number of RDH structures in these mutants (Supplementary Figure S7). These observations suggested that these two mutant RNase H proteins were bound to the RDH structures *in vivo* but did not cleave the RNA present in these structures as they do *in vitro* [54], and also might not allow Rho to remove them by blocking the latter’s access to the RDHs *in vivo*.

**Figure 6:**
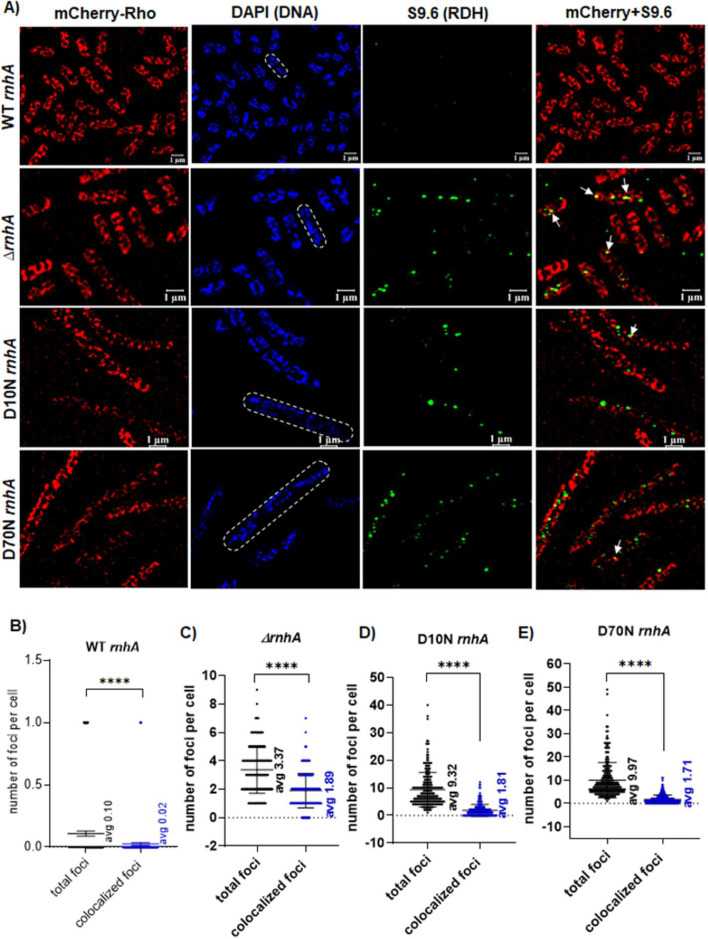
Colocalization of Rho with the RDH structures in the presence of rnh point mutants. (**A**) Super-resolution microscopy images of the *E. coli* MC4100 cells expressing WT mCherry-Rho (red) in the presence and absence of RNase H and the presence of D10N and D70N RNase H mutants. In these experiments, RDH structures were probed with immunofluorescence using mS9.6 antibody DNA was probed with DAPI staining in a similar way as described in Figure 4. Colocalizations of Rho and RDH structures are indicated by white arrowheads in the merged images as yellow spots. The number of S9.6 foci and the colocalized foci per cell (*n* ≥ 200) are shown as scattered plots in the case of WT *rnhA* (**B**), ∆*rnhA* (**C**), D10N *rnhA* (**D**), and D70N *rnhA* (**E**). The t-test (unequal variance) was used for the determination of statistical significance (**** indicated *P* value <0.0001). The error bar represents the SEM. RDH, RNA:DNA hybrid.

To further confirm the blockage of access of Rho to RDH by the D10N and D70N RNase H mutants, we monitored the colocalization of Rho with the RDH structures in the presence of RNase H point mutants in a similar way that is described in Figure 4 by IF with mS9.6 antibody of the mCherry-Rho expressing *E. coli* MC4100 cells (Figure 6A). The highly elongated D10N and D70N expressing strains were very sick, so it was difficult to perform genetic manipulations on these strains. To reduce a genetic manipulation step (e.g., P1 transduction), we did not remove the chromosomal *rho* before introducing the mCherry-Rho (unlike what is described in Figure 4). We acquired the mCherry-Rho images in the presence of the WT Rho expressed from the chromosome, which led to the formation of Rho with mCherry-tagged and untagged subunits, and the signal intensities of mCherry were reduced, giving rise to higher background noises in some of the images. When WT RNase HI was present, the number of RDH foci was much less (Figure 6B). The number of RDH foci increased from ~3 per cell when RNase HI was absent (Figure 6C) to ~9–10 per cell in the presence of D10N and D70N mutants of RNase HI (Figures D and E). However, out of these ~10 RDH foci, only ~2 per cell were found to be colocalized with the Rho foci, giving rise to yellow puncta (Figure 6D and E). To further validate these visual observations, we quantitatively analyzed the fraction of colocalized spots using the Zen 3 software built into the microscope. The bar graphs (Supplementary Figure S9E) showed the fraction of colocalized foci of Rho with RDH in the strains expressing the D10N and D70N RNase HI reduced to ~20% from 72% that was observed in the absence of any RNase HI. We then created scattered plots of the green (RDH signal) against the red (Rho signal) spot intensities to estimate the Pearson correlation coefficient (P) of the probability of colocalization of these two signals in the strains expressing the RNase HI mutants (Supplementary Figure S9). We observed that these two signal intensities in the RNase HI mutant strains are negatively correlated (Supplementary Figure S9), and hence, we concluded that the mCherry-Rho and S9.6-labeled RDH spots in these two strains do not colocalize in a significant manner. These observations confirm that RNase H mutants block the access of Rho to the RDH structures, leading to a deleterious effect on the cell, which is reflected in the severe growth defect of these mutants (Figure 5A).

### Rho unwinds RDHs in a *rut* site-dependent manner via its helicase activity and also binds directly to RDHs *in vitro*

**Figure 7:**
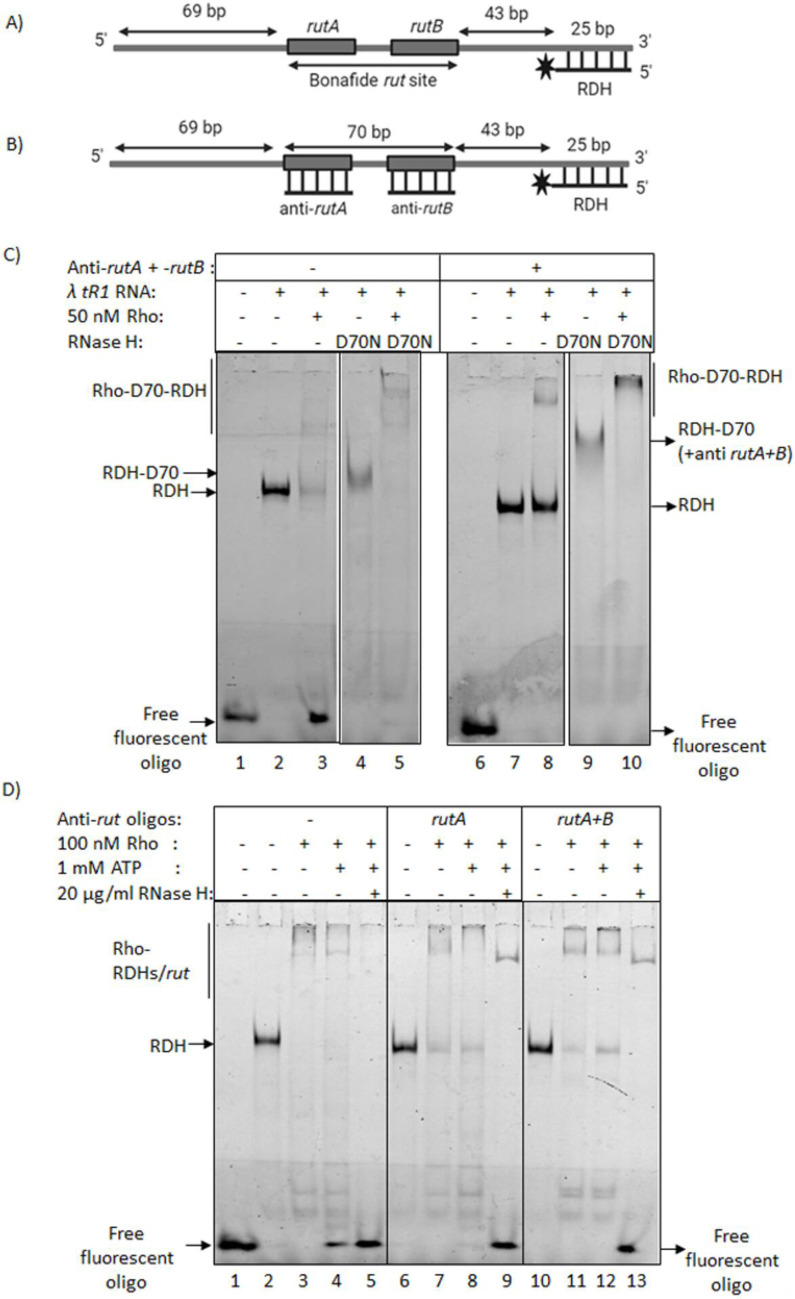
*In vitro* Rho-mediated unwinding of fluorescent DNA-labeled RDH. (**A,B**) Cartoons showing the *in vitro* synthesized 207nt *lt_R1_* RNA template with the *rutA* and *ruB* sequences. A 25-nt 3′-fluorescent-tagged primer (RS588) is shown to hybridize towards the 3′-end of the RNA template to form the requisite RDH structure. The antisense oligos against the *rut* sites are also indicated. (**C**) Gel-shift assays of the fluorescent oligo in the presence of the RNA template, as well as Rho and RNase H proteins, as shown in different lanes. The samples were run on a native 6% PAGE and were scanned by the phosphorimager. The concentration of the RNA template was 50 nM, and the fluorescent oligo was 25 nM. When required, 200 nM of anti-rut oligos was used. The concentrations of Rho and the mutant RNase H were 50 nM and 20 mg/ml, respectively. 1 mM ATP was present in the reactions. (**D**) Binding assays of Rho with the RNA template having an RDH made of a fluorescent oligo, as described in (**A**), in the presence and absence of anti-Rut oligos, are shown in a native 6% PAGE. The lanes were indicated, and RNase H was added to the Rho-RDH binding reaction at 37°C for 10 min. The samples were run on a 6% native PAGE, and the gel was scanned by the phosphorimager. The concentration of Rho was 100 nM. The reaction was set up with or without 1 mM hydrolyzable ATP. The concentration of anti-Rut oligo was 200 nM, and RNase H was 20 µg/ml. The binding and unwinding assays were performed in an RNase H reaction buffer (200 mM Tris-HCl, pH 7.8, 400 mM KCl, 80 mM MgCl_2_, and 10 mM DTT). RDH, RNA:DNA hybrid.

To determine the mechanism of action of Rho to remove the RDH structures, we monitored the RDH binding and unwinding activities of Rho in a purified system in the absence and presence of WT and mutant (D10N and D70N) RNase H. We synthesized a 207 nt RNA template having the *lt_R1_* Rho-dependent terminator region (with the Rho-utilization sites, *rutA* and *rutB,* indicated in Figure 7A). We used a 3′-Fluorescein-labeled 25nt DNA oligo complementary to the 3′ region of the RNA template to form an RDH structure as shown in Figure 7A. In these assays, upon the unwinding of the RDH by RNase H-mediated cleavage or due to the helicase action of Rho, the fluorescent oligo will be released from the RDH structure and will migrate as a free oligo in native PAGE. If the RDH is bound to Rho or RNase H under certain conditions, gel-shifted bands of the RDH-Rho/RNase H complexes will be visible.

Using this experimental set-up, we observed that WT RNase HI in the presence of MgCl_2_ released the free fluorescent oligo using its cleavage activity on the RNA of the RDH (Supplementary Figure S10). In the presence of the RNase H mutants (Lanes 3–10, Supplementary Figure S10) and the WT RNase HI in a MgCl_2_-depleted buffer (Lanes 15–18, Supplementary Figure S10), we observed the formation of RNase HI-bound gel-shifted RDH complexes.

When Rho was added to the RDH structure formed on the RNA, a major fraction of the RDH band disappeared with the release of the free fluorescent oligo, indicating the Rho-unwinding activity to remove the RDH (left panel of Figure 7C, compare Lanes 2 and 3). However, this Rho-mediated release of the oligo was not observed when the RDH was incubated with the RNase H mutant D70N; instead, a supershifted band presumably of the RDH-D70N-Rho ternary complex was observed (Figure 7C, Lanes 4 and 5). These data suggested that the tightly bound D70N RNase H-RDH complex most likely blocked the 5′ to 3′ Rho helicase activity at the RDH structure.

Rho exerts 5′ to 3′ RNA helicase function following the binding to specific Rho-binding sites, the *rut* sites [58–60]. We next checked the *rut*-site binding dependence of Rho to remove the RDH structure in the above-described assays. As shown in Figure 7B (presence of three RDHs on the RNA template), we blocked the *rutA* and *rutB* sites of the RNA template by adding corresponding anti-sense oligos, following which we performed the Rho helicase assay on this template. We observed that Rho was not able to release the fluorescent oligo from the RDH (Lanes 7 and 8 of the right panel of Figure 7C). In the presence of D70N RNase H and anti-*rut* oligos, Rho formed a supershifted ternary complex with D70N and RDH structures (Figure 7C, Lane 10). D70N formed complexes with all three RDHs (Figure 7B) to form a gel-shifted band that migrates slower (Figure 7C, lane 9) than when D70N formed a complex with the single RDH (Figure 7C, Lane 4). We concluded that the presence of *rut* site sequences on the RNA template upstream of the RDH structures is an *a priori* requirement for Rho to unwind and remove these structures. Rho is also capable of forming ternary complexes with D70N RNase H and RDH structures in the absence of its helicase activity.

Since Rho can form ternary complexes with RDH and the RNase H mutants, it is possible that Rho could directly bind to RDH structures independent of its helicase activity or when the *rut* sites upstream of these structures are inaccessible. We used the same experimental setup as described in Figure 7A and B. In the presence or absence of the oligos complementary to *rutA* and *rutB* and that with the fluorescent tag, the RNA templates with either one (in the absence of both anti-*rutA* and *rut*B oligos) or two (in the absence of *anti-rutB* oligo) and three (in the presence of all the three oligos) RDH structures will be formed. We monitored Rho-binding to the RNA templates with these various RDH structures. We observed the formation of gel-shifted complexes in the presence of higher concentrations of Rho (Figure 7D), both in the absence and presence of the anti-*rut* oligos. The gel-shifted complexes of Rho with RNA templates with two or three RDH structures are likely to be Rho-RDH binary complexes because, under these conditions, there are no bona fide Rho-binding sites on this RNA template (Figure 7D, Lanes 7, 8, 11, 12). In the absence of anti-Rut oligos, the binary complexes are likely to be Rho-*rut* complexes (Figure 7D, Lanes 3, 4). We also observed that in all the cases, RNase HI was able to exert its function of cleaving the RNA component of the RDH and releasing the fluorescent oligo (Figure 7D, Lanes 5, 9, 13). These data suggested that Rho-bound RDHs did not hinder the RDH-cleavage function of RNase HI. It is possible that this Rho-binding of RDH could have functional relevance in augmenting the function of RNase H *in vivo* when both are present in the cell. However, we cannot rule out that Rho-RDH binary complex formation is only an *in vitro* phenomenon, and we do not have any evidence to claim that this direct RDH-binding by Rho is physiologically relevant.

## Discussion

The bacterial transcription terminator Rho is an RNA helicase that uses energy from ATP hydrolysis to execute its motor function to unwind RNA secondary structures as well as RDHs *in vitro* [34,58,59,61]. Rho can exert this helicase function independently of its transcription termination function *in vitro* when its RNA substrate is not a part of the transcription EC. Hence, Rho might have an important role, like other RNA helicases *in vivo* in addition to being a transcription terminator. Here, we have provided genetic, biochemical, and cell biological evidence to prove that Rho is involved in removing the deleterious RDHs *in vivo,* which are formed on the RNA that may or may not be part of the EC. Here, we showed that the Rho PBS mutants defective in RNA-binding and helicase function are synthetically lethal in the absence of RNase HI but not in the absence of RNase HII (Figure 1). The Rho-PBS mutant exhibited increased plasmid-concatemer and plasmid copy number, altered cell morphology, and was highly susceptible to DNA damaging agents in a *rnhA^-^* strain (Figure 2 and S2) and induced accumulation of RDHs (Figure 3), which suggested severe defects in the RDH removal process when both RNase HI and WT Rho are absent, indicating functional complementation of RNase HI function by Rho. We demonstrated the localization of a significantly higher number of Rho molecules near the RDHs *in vivo* when RNase HI was absent (Figure 4). Catalytically inactive mutants of RNase HI (D10N and D70N) that bind to the RDH blocked the entry of Rho to the RDH, leading to cell death, further reinforcing the role of Rho in the removal of deleterious RDHs in the absence of RNase HI (Figures 5 and 6). Under *in vitro* conditions, in a purified system, Rho was capable of binding to the RDHs and unwinding them in a *rut*-site-dependent manner (Figure 7). Therefore, we concluded that in the absence of RNase HI, Rho, by its RNA-dependent helicase activity, is capable of unwinding RDHs in a *rut*-site-dependent manner *in vivo,* which prevents the formation of these structures on RNA as well as disassembles them. These results established that Rho can function as a bona fide RNA helicase independent of its role as a transcription terminator, and its direct RDH binding property *in vitro* led us to speculate that both Rho and RNase HI could have a functional synergy while acting on the RDHs *in vivo*.

Classically, Rho-dependent transcription termination is viewed as an important step in gene regulation, controlling the expression of close to one-third of operon transcription in a dividing *E. coli* culture [20,21,62,63]. This leads to Rho’s involvement as a terminator in the regulation of transcription-translation coupling [24,64], control of riboswitch action [28,30,65], suppression of pervasive and antisense transcription [20,21,66], silencing and maintaining prophages [62], suppressing the toxin–antitoxin modules of cryptic prophages [67], and recycling stalled RNA polymerases at the DNA lesions [40]. Our results here indicate that the removal of the deleterious RDHs in the absence of RNase HI is executed by Rho by its helicase function in a terminator-independent manner. We argue that the removal of the RDHs of the R-loops by Rho [37] may not involve the latter’s transcription termination function. We speculate that in addition to the suppression of anti-sense transcription by Rho [20,21], the putative RDH formed by these anti-sense transcripts could be removed by the Rho helicase function, independent of its termination function.

Rho is an SF-V type hexameric RNA-dependent helicase [61], and its RNA:RNA and RNA:DNA unwinding activities through its motor function are well established in *E. coli* and other bacteria [34,39,60,68,69]. Our results suggest that Rho could function as an RNA helicase *in vivo,* like other *E. coli* RNA helicases, such as Dead-box-, DEAH/RHA-, and Ski2-family helicases [70] RA. However, they are involved in RNA remodeling and chaperoning. However, since Rho prefers C-rich G-poor sequences (*rut* sites) on RNA to bind, the helicase function would be contingent upon the existence of these types of sequences upstream of the RNA secondary structures that are required to be remodeled by Rho. In support of this proposition, we have recently observed that Rho is capable of complementing the function of the RNase R, which is a helicase and also a 3′-5′ ribonuclease (unpublished observation). It should be noted that the Rho helicase is not capable of removing any roadblock that it faces during its translocation along the RNA, as it was observed to be blocked by the stable RNase HI mutant (D10N and D70N)-RDH complexes. We speculate that cells use the Rho helicase function as a backup mechanism to execute the very important job of removing deleterious RDHs or proper folding of RNA under various conditions when other helicases fail to execute.

*Does Rho evolve primarily to function as an RNA helicase and not as a transcription terminator?* Our high-resolution microscopy data [53] (Supplementary Figure S3) revealed that the mCherry-Rho molecules are distributed along the inner membrane at the poles of the *E. coli* log phase culture and not colocalized with the transcription machinery on the bacterial chromosome. This suggests that the majority of the Rho molecules do not reside near the transcription machinery in the dividing cells. Since ribosomes function in a membrane-bound form [71], RNA degradosome complexes reside on the membrane [72], and many other metabolic processes take place on the membrane in *E. coli*, we propose that the membrane-bound Rho molecules function primarily as RNA helicase to exert RNA remodeling required for ribosome assembly and RNA chaperoning into RNA degradosome complexes that are located in the vicinity.

## Materials and methods

### Materials

All antibiotics except BCM used in this study were purchased from Sigma. BCM was from SantaCruze. The *E. coli* Ribonuclease H enzyme was purchased from Epicentre. All primers used in this study were from Eurofins Genomics India Pvt. Ltd., and are listed in Supplementary Table S1. All the plasmids used during the study are listed in Supplementary Table S2. S9.6 anti-RDH monoclonal antibody (MABE1095) was purchased from Merck, India. Agarose was from Lonza. The DotBlot apparatus used for immunoblotting was from Bio-Rad. The antifading agent and DAPI (4,6-diamidino-2-phenylindole) were obtained from Thermo Fisher Scientific. Bovine serum albumin (BSA) was purchased from Amresco. The molecular biology grade buffers and enzymes were obtained from NEB. The dNTPs mixture was obtained from Thermo Fisher Scientific. PBS for microscopy was from Gibco. The Taq polymerase for the *in vitro* experiment template preparation was purchased from Roche. The RNA template for *in vitro* hybrid formation was prepared using the CellScript RNA preparation kit.

### Bacterial strains

The bacterial strains used in this study are listed in Supplementary Table S1. *E. coli* MG1655 and MC4100 were used as parental strains for different modifications in the study. Since Rho is an essential gene, an exogenous copy was provided through IPTG-dependent shelter pHYD1201 in RS1305 (*E. coli* MG1655 ∆*rho*). RS1309 (*E. coli* MG1655 ∆*rac* ∆*rho*) was generated from RS1305 (*E. coli* MG1655 ∆*rho*) by deletion of the *rac* gene by P1 transduction. When required, *rnh* genes (*rnhA*, *rnhB*, and *rnhAB*) that code RNase HI and HII were deleted with P1 transduction from RS1309 (having pHYD1201). The deletion of *rnh* genes in each case was confirmed by PCR amplification of that region of the chromosome. To check the combinatorial effects of the deletion of *rnhA* in the presence of different Rho mutants, we used *E. coli* MC4100 having WT chromosomal Rho (RS257) and Y80C Rho (RS1714) in the chromosome. These strains were made ∆*rnhA* by the P1 transduction.

### Synthetic growth defect assessment

To examine the synthetic growth defects of the *rnh* genes (*rnhA, rnhB*) with the Rho mutants, the former genes were deleted individually or together by the P1 transduction in RS1309 carrying pHYD1201 expressing the WT Rho. Strains that carry a deletion of both the Rho and each of the *rnh* were transformed with plasmid pCL1920 expressing either WT or mutant Rho and then cured of the shelter plasmid, pHYD1201. To assess the growth defects of strains carrying a deletion of *rnh* in combination with mutant Rho, overnight cultures of these strains were sub-cultured, and the growth curves were obtained by growing in microtiter plates and using a SpectramaxM5 microtiter plate reader (Molecular Devices, U.S.A.) at OD_600nm_. These strains were also streaked on LB plates to assess the synthetic defects.

### Growth defects in the presence of DNA-damaging agents

The mid-log cultures of MC4100 were serially diluted, spotted on LB agar plates, and incubated at 25°C for 30 min. These plates were then exposed to various UV doses (0, 5, 10, 20, 30, and 40 J/m^2^) of 254 nm UV light in a UV cross-linker (CX-2000 UV cross-linker, Ultra-Violet Products Ltd., Cambridge, UK). After UV exposure, plates were covered with aluminum foil and incubated overnight at 37°C. The fraction of surviving colonies relative to the colonies grown on the unexposed plates was calculated and plotted against the UV doses [19]. Similarly, a Mitomycin C sensitivity assay was performed for MC4100 derivatives using different mitomycin concentrations ranging from 0, 0.25, 0.5, 0.75, and 1 μg/ml on the LB plates [40].

### Plasmid copy number and concatemer analyses

To assess the plasmid copy number of pBR322, we transformed the plasmid to RS257, RS2105 (*rnhA^-^rho^+^*), RS1714 (*rnhA^+^/*Y80C *rho*), and RS2106 (*rnh^-^/*Y80C*rho*). pBR322 was isolated from mid-log phase cultures. An equal volume of isolated plasmid was run on a 1% agarose gel for 90 min, and images were captured using gel image capture (iGene Labserve, India). Concatemer formation in the mutant strains was analyzed following the previously published report [50].

### Cell morphology and nucleoid morphology analysis

Mid-log phase (OD_600nm_ ~0.4 at 37°C) cultures were centrifuged at 4000 rpm for 10 min and washed at least 3 times with 1X PBS. The pellets were fixed with 4% paraformaldehyde for 20 mins on ice, followed by three washes with 1X PBS, followed by staining with DAPI (5 µg/ml). Finally, the pellets were washed again three times with PBS, resuspended, and 5 µl samples were mounted on a slide. Slides were observed using a confocal microscope (confocal LSM 900), and the size of the cells was estimated from 500 cells using ImageJ software.

### Immunoblot of RDH

The *in vivo* RDH was detected by an immunoblotting technique using the mS9.6 (monoclonal S9.6, Merck, India) mouse antibody raised against RDH [38]. Briefly, the total genomic DNA was isolated from the strains by the phenol-chloroform isoamyl alcohol extraction method. Then, 1 μg of isolated DNA from each culture was immobilized on Hybond N^+^ nylon membrane (Amersham Bioscience) using the Bio-Dot® Microfiltration Apparatus (BioRad, U.S.A.) and cross-linked by UV cross-linker at 1200 J/m^2^. When RNase H treatment was required, the DNA was treated with 0.01 U of RNase H enzyme at 37°C for 20 min before blotting. After UV cross-linking, the membrane was first incubated with a 1:10,000 dilution of the mS9.6 antibody at 4°C overnight and then with a secondary HRP-conjugated antibody for 45 min. The immunoblots were detected with the help of a chemiluminescence kit (ThermoFisher Scientific, U.S.A.), and the signals were quantified by ImageQuant software.

### IF microscopy

The presence of RDHs in the cell and their colocalization with Rho were detected using IF microscopy. In these experiments, Rho was tagged to mCherry [53], and the RDHs were detected using the mS9.6 antibody conjugated to a fluoro-labeled (either rhodamine or Alexa Fluor 488) secondary antibody. For the detection of RDHs only, a rhodamine-labeled (red) secondary antibody was used, whereas to detect the Rho-RDH colocalization, an Alexa Fluor 488-labeled (green) secondary antibody was used. Mid-log phase culture (~0.3 OD_600_) was harvested for all the microscopy experiments. For the Rho-RDH colocalization assays, a mid-log phase culture of 0.3 OD_600nm_ was pelleted and treated with 1% formaldehyde for 30 seconds for *in vivo* cross-linking at room temperature, followed by a 100 mM glycine treatment for 30 min at 4°C to stop the crosslinking reaction. Then, cells were harvested and incubated with ice-cold 100% methanol for 40 min on ice, followed by twice 1X PBS washes. For further fixation, the cells were mixed with 4% paraformaldehyde on ice for 10 min. Finally, after washing the pellet with PBS twice, it was resuspended in the same buffer and applied to the polylysine-coated slides to form a smear (SIGMA Aldrich P8920). After semi-drying, the smear on the slides was treated with 20 µL of 2 mg/ml of lysozyme solution for 5 min at room temperature, then washed with PBS and blocked with 2% BSA for 1 h. at room temperature, following which the smear was first treated with 1:1500 dilution of mS9.6 mouse antibody for 2 h, and then with Alexa fluor 488-labeled secondary anti-mouse antibody for 45 min in the dark followed by three washes with PBS in each stage. For RDH detection experiments, a rhodamine-labeled secondary antibody was used. Finally, the smear was stained with DAPI and visualized in an ELYRA 7 super-resolution SIM microscope. The images were taken with Andro1, AxioObserver Microscopy software. The objective lens was a Plan-Apochromat 63 x/1.4 Oil DIC M27 with a laser emitting at 561 nm for mCherry fluorescence detection and at 405 nm for DAPI fluorescence detection. The exposure time was 90 ms, and the depth of focus was 0.87 μm and 0.63 μm for mCherry and DAPI, respectively. Images were post-processed using Zen v3 software. The constant signal color contrast was maintained by reducing the noise/background, and the images were exported in the *.tiff format.

### Purification of RNase H protein and its mutants

RNase H proteins were purified by Ni-NTA column as described previously by Lee H, et al. [73]. The WT RNase H gene from *E. coli* was cloned in a pET21b vector containing a 6X His tag at the C-terminal using *XhoI* and *NdeI* restriction sites. The aspartic acid residues at 10^th^ (D10N) and 70^th^ (D70N) positions were changed to asparagine by site-directed mutagenesis (SDM). The protein was purified using Qiagen Ni-NTA agarose beads and stored in a buffer containing 50 mM KCl, 10 mM Tris-Cl (pH 7.5), 0.1 mM EDTA, 1 mM DTT with 50% glycerol at −20°C.

### Preparation of RDH substrate and gel-shift assays

The RNA with the Rho-dependent terminator, *lt_R1_* sequence, was synthesized from the DNA template where this terminator sequence was fused downstream of a T7-f10 promoter using the T7-FlashScribe Transcription kit. For RDH preparations, 25 nM of 3′-fluorescein-labeled 25 nt RS588 DNA oligo was mixed with 50 nM of 207 bp *lt_R1_* RNA in annealing buffer containing 10 mM Tris-Cl (pH 7.6), 50 mM NaCl, and 1 mM EDTA. The annealing was performed using a slow cooling method described earlier [74]. This template with the RDH was used as a substrate for all *in vitro* experiments. When required, anti-rutA and rutB oligos were added to this template. All the binding and RDH unwinding reactions were performed in RNase H reaction buffer containing 20 mM Tris-Cl (pH 7.8), 40 mM KCl, 8 mM MgCl_2_, and 1 mM DTT at 37°C for 10 min. For the Rho-dependent unwinding of the RDH from this template, 1 mM hydrolyzable ATP and 50 nM or 100 nM Rho in RNase H reaction buffer in the presence or absence of the anti-rut oligos were added. Reactions were performed at 37°C for 10 min. To block the *rut* sites, 200 nM of anti-*rutA* and anti-*rutB* oligos were used. The reaction mixtures were loaded onto 6% native PAGE in a cold room. The gel images were scanned using a Typhoon phosphorimager in the fluorescein emission range of 514 nm.

### Quantitative and statistical analysis of microscopic data

The cell length distribution (Supplementary Figures S4 and S8) was subjected to Gaussian analyses using SIGMAPLOT 15. From the distribution plots, 5% outliers were ignored, and the areas around the peaks of the Gaussian Distribution curves were considered for the calculations of the median of cell length. Wherever described, the number of S9.6 spots per cell per median µm was calculated by dividing the foci number by the median values of the cell length. To estimate the statistical significance of the occurrence of the RDH foci (S9.6 spots), the probability of the number of foci per cell or median µm for each cell was plotted in a Poisson distribution plot (Figures 3, 5, S4). These plots were generated using a dedicated Python in-built script with the PD library where the probability of event occurrence was calculated by the software itself using the formula: P(*X* = *k*) = *e*^- λ^λ^k^ / k! where *X* = random variable following Poisson distribution, *k* is the number of times the event occurred, e is Euler’s constant (~2.718), *λ* is the average number of times an event occurs (the Poisson distribution rate), and is the *!* is the factorial function.

At first, we estimated the colocalization of the mCherry-Rho and RDH spots by visual inspection of the occurrence of yellowish spots from ~250 cells and plotted them as scattered using GraphPad Prism 8.0 (Figures 4B,C,6B-E). To validate these data more quantitatively, we quantified the fraction of RDH foci colocalized with Rho using Zen3 image analysis software. The results were represented as bar graphs created with SIGMAPLOT 15 (Figure 4D, S9E). Foci from ~100 cells were considered for the calculations.

We employed Pearson coefficient analysis to estimate the statistical significance of the colocalizations of mCherry-Rho and the RDH spots in Figures 4 and 6. We plotted the two signals emanating from the mCherry-Rho (red) and the S9.6 (green) fluorescence in a scattered plot to calculate the colocalization correlation coefficient between the two signals using Zen3 software built into the super-resolution microscope (Supplementary Figures S6 and S9). The four quadrants of the scatter plot represent the intensity of red color (Q1, mCherry signal), green color (Q2, S9.6 signal), colocalized region (Q3), and background intensity (Q4). Intensities of each colored spot (pixels) were obtained from ~100 cells in each case. From these scattered plots, we calculated the PCC between mCherry-Rho and RDH spots to estimate the statistical significance of the colocalization by Zen3 software and plotted the values using SIGMAPLOT 15 (Figure 4E and S9F).

## Supplementary material

Online supplementary tables

Online supplementary figure S1

Online supplementary figure S2

Online supplementary figure S3

Online supplementary figure S4

Online supplementary figure S5

Online supplementary figure S6

Online supplementary figure S7

Online supplementary figure S8

Online supplementary figure S9

Online supplementary figure S10

## Data Availability

All the raw data used to prepare the final figures or tables are available upon request. This manuscript does not contain any dataset that must be deposited in any data bank.

## Open Access

This article has been published open access under our Subscribe to Open programme, made possible through the support of our subscribing institutions, learn more here: https://portlandpress.com/pages/open_access_options_and_prices#conditional

## Abbreviations

EC: elongation complex
PBS: primary RNA-binding site
RDH: RNA:DNA hybrid.
SBS: secondary RNA-binding site
WT: wildtype
