# Correction: the bacterial transcription terminator, Rho, functions as an RNA:DNA hybrid (RDH) helicase *in vivo*

**DOI:** 10.1042/BCJ-2025-3089_COR

**Published:** 2025-11-19

**Keywords:** fluorescence microscopy, Rho, RNase H, RNA:DNA hybrid, rut-site, synthetic lethality

The authors of the article “The bacterial transcription terminator, Rho, functions as an RNA:DNA hybrid (RDH) helicase *in vivo*” (DOI: 10.1042/BCJ20253089) would like to correct the affiliation of the first author, Ankita Bhosale.

The correct affiliation for Ankita Bhosale is Research Scholar, Manipal Academy of Higher Education, Manipal, Karnataka, India.

