# Supplementary material for: The bacterial transcription terminator, Rho, functions as an RNA:DNA hybrid (RDH) helicase in vivo
Source: Biochem J. 2025 May 26;482(11):655–74. doi: 10.1042/BCJ20253089 (PMC12203952; doi:10.1042/BCJ20253089)
Supplement: Online supplementary tables [file BCJ-482-11-BCJ20253089-s001.pdf]

Table S1. Oligos were used in this study.

Primer	Description
RS2079	CAACCGTATGGAGTTGATGGC; upstream oligo for *rnhA* gene used for RT-qPCR
RS2080	TCAAGACGTTGCCAGAGATCG; downstream oligo for *rnhA* gene used for RT-qPCR
RS2365	GCGCAAGCTTGGTTTCGGTATCGAGAACGAT; upstream oligo with *HindIII* site for cloning of *E. coli rnhA* in pOK12 and pCL1920 vector
RS2366	CGCGGAGCTCTTAAACTTCAACTTGGTAGCC; downstream oligo with *SacI* site for cloning of *E. coli rnhA* in pOK12 and pCL1920 vector
RS2367	GCGCGCGCCATATGCTTAAACAGGTAGAAATT; upstream oligo with *NdeI* site for cloning of *E. coli rnhA* in pET 21b vector
RS2368	GCGCGCCTCGAGAACTTCAACTTGGTAGCCTGT; downstream oligo with *XhoI* site for cloning of *E. coli rnhA* under in pET 21b vector
RS2369	AAACAGGTAGAAATTTTCACCAATGGTTCGTGTCTGGGCAATCCA; upstream oligo for making D10N *rnhA* point mutant by SDM
RS2370	TGGATTGCCCAGACACGAACCATTGGTGAAAATTTCTACCTGTTT; downstream oligo for making D10N *rnhA* point mutant by SDM
RS2371	TGCGAAGTCATTTTGAGTACCAACAGCCAGTATGTCCGCCAGGGT; upstream oligo for making D70N *rnhA* point mutant by SDM
RS2372	ACCCTGGCGGACATACTGGCTGTTGGTACTCAAAATGACTTCGCA; downstream oligo for making D70N *rnhA* point mutant by SDM
RS588	AAG CTT CGC CCG TGT CCC TCT CGA T; 3'-Fluorescein labeled oligo compliment to 3' end of λtR1 RNA
RS2442	TTAATACGACTCACTATAGGGAGATCACTATAAACGCTGATGG; upstream oligo to generate λtR1 DNA template fused with upstream T7 promoter sequence
RS2440	AAGCTTCGCCCGTGTCCCTCTCGATTCCTTAGATAACAATTGATTGAATG; downstream oligo to generate λtR1 DNA template downstream fused with anti-sense fluorescein labeled sequence
RS367	GGAATGTGTAAGAGCGGGGTTATTTATGC; antisense to *rutA* site of λtR1 RNA
RS368	CACCATAGGTGTGGTTTAATTTG; antisense to *rutB* site of λtR1 RNA

Table S2. Plasmids used in this study:

Plasmid name	Description	Reference
pHYD1201	*rho* subcloned in pAM 34 at *HindIII-SalI* site from pHYD567 (pMB9; *Amp*^{r}, IPTG dependent replicon)	[1]
pRS317	*E. coli* Rho cloned in pCL1920 (*Spec*^{r})	[2]
pRS1109	Y80C Rho in pCL1920 (*Spec*^{r})	[2]
pRS966	P103L Rho in pCL1920 (*Spec*^{r})	[3]
pRS1106	G324D Rho in pCL1920 (*Spec*^{r})	[2]
pRS725	N340S Rho in pCL1920 (*Spec*^{r})	[2]
pRS2264	Rho-mCherry tag with overlap extension PCR and cloned under *rho* promoter at N-terminal end in pCL1920 (*Spec*^{r})	[5]
pRS2331	*E. coli rnhA*, along with its promoter, cloned in pOK12 plasmid at the sites *HindIII* and *sacI* (*Kan*^{r})	This study
pRS2332	pOK12 *rnhA* D10N by SDM of pRS2331 (*Kan*^{r})	This study
pRS2333	pOK12 *rnhA* D70N by SDM of pRS2331 (*Kan*^{r})	This study
pRS2305	*E. coli rnhA* cloned in pET21b at *XhoI* and *NdeI* sites under T7 promoter (*Amp*^{r})	This study
pRS2306	pET21b *rnhA* D10N by SDM of pRS2305 (*Amp*^{r})	This study
pRS2307	pET21b *rnhA* D70N by SDM of pRS2305 (*Amp*^{r})	This study
pRS604	T7A1-λT_{R1} fragment cloned at *HindIII* site of pRS22 (*Amp*^{r})	[4]
pRS566	pBR322 empty vector in DH5α (*Amp*^{r})	
pRS575	pCL1920 empty vector in in DH5α (*Spec*^{r})	
pRS1103	Pcp20 plasmid in DH5α (*Cam*^{r})	From J Gowrishankar

References

1 Harinarayanan, R. and Gowrishankar, J. (2003) Host factor titration by chromosomal R-loops as a mechanism for runaway plasmid replication in transcription termination-defective mutants of Escherichia coli. *J. Mol. Biol.* **332**, 31–46 https://doi.org/10.1016/S0022-2836(03)00753-8

2 Chalissery, J., Banerjee, S., Bandey, I. and Sen, R. (2007) Transcription Termination Defective Mutants of Rho: Role of Different Functions of Rho in Releasing RNA from the Elongation Complex. *J. Mol. Biol.*, Elsevier Ltd **371**, 855–872 https://doi.org/10.1016/j.jmb.2007.06.013

3 Valabhoju, V., Agrawal, S. and Sen, R. (2016) Molecular basis of NusG-mediated regulation of Rho-dependent transcription termination in bacteria. *J. Biol. Chem.*, American Society for Biochemistry and Molecular Biology Inc. **291**, 22386–22403 https://doi.org/10.1074/jbc.M116.745364

4 Dutta, D., Chalissery, J. and Sen, R. (2008) Transcription termination factor Rho prefers catalytically active elongation complexes for releasing RNA. *J. Biol. Chem.*, Â© 2008 ASBMB. Currently published by Elsevier Inc.; originally published by the American Society for Biochemistry and Molecular Biology. **283**, 20243–20251 https://doi.org/10.1074/jbc.M801926200

5. Jain, S., Behera, A., and Sen, R. (2025). DNA binding of an RNA helicase bacterial transcription terminator. Biochem. J., Portland Press Limited 482, 1–15 https://doi.org/10.1042/BCJ20240452
