# Supplementary material for: The bacterial transcription terminator, Rho, functions as an RNA:DNA hybrid (RDH) helicase in vivo
Source: Biochem J. 2025 May 26;482(11):655–74. doi: 10.1042/BCJ20253089 (PMC12203952; doi:10.1042/BCJ20253089)
Supplement: Online supplementary figure S1 [file BCJ-482-11-BCJ20253089-s002.pdf]

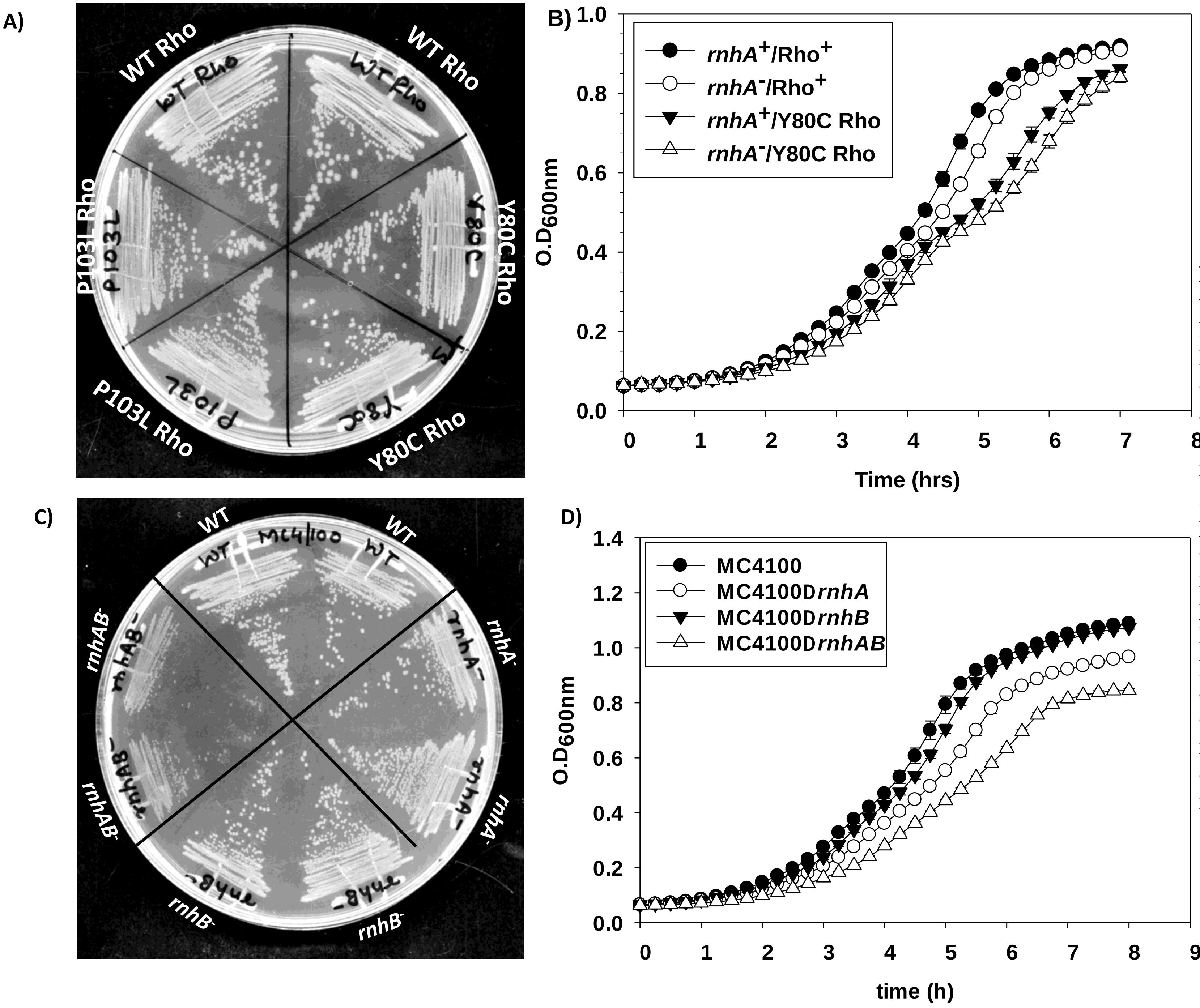

Figure S1. A) LB plate shows the side-by-side streaking of Rho mutants (Y80C and P103L) in the presence of WT chromosomal Rho, WT *rnhA*, and *rnhB*. B) Growth curves of MC4100 strains with indicated genotypes of *rho* and *rnh*. Mean growth curves are plotted and the error bars were obtained from three independent colonies. C) LB plates show the growth of MC4100 strains in the presence and absence of *rnhA* and *rnhB*. D) Growth curves of the same strains as indicated. Error bars were obtained from three independent measurements.
