# Supplementary material for: The bacterial transcription terminator, Rho, functions as an RNA:DNA hybrid (RDH) helicase in vivo
Source: Biochem J. 2025 May 26;482(11):655–74. doi: 10.1042/BCJ20253089 (PMC12203952; doi:10.1042/BCJ20253089)
Supplement: Online supplementary figure S2 [file BCJ-482-11-BCJ20253089-s003.pdf]

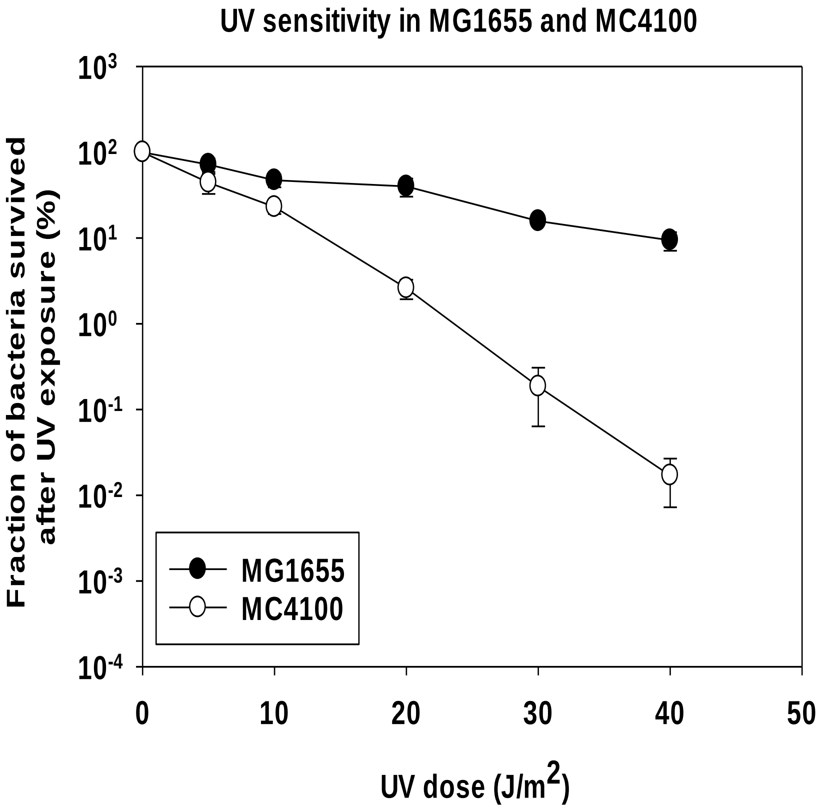

Figure S2. **UV sensitivity comparison between MG1655 and MC4100.** Plots showing the effects of different UV doses (J/m^2) on the survival of the MG1655 and MC4100 expressed as fractions of bacteria that survived after treatments. The fractions of survived bacteria were calculated by the formula: [(total no. of colonies from the plates after UV treatment)/ (total number of colonies from corresponding to untreated plates)]*100. The SEM was calculated from three independent experiments.
