# Supplementary material for: The bacterial transcription terminator, Rho, functions as an RNA:DNA hybrid (RDH) helicase in vivo
Source: Biochem J. 2025 May 26;482(11):655–74. doi: 10.1042/BCJ20253089 (PMC12203952; doi:10.1042/BCJ20253089)
Supplement: Online supplementary figure S3 [file BCJ-482-11-BCJ20253089-s004.pdf]

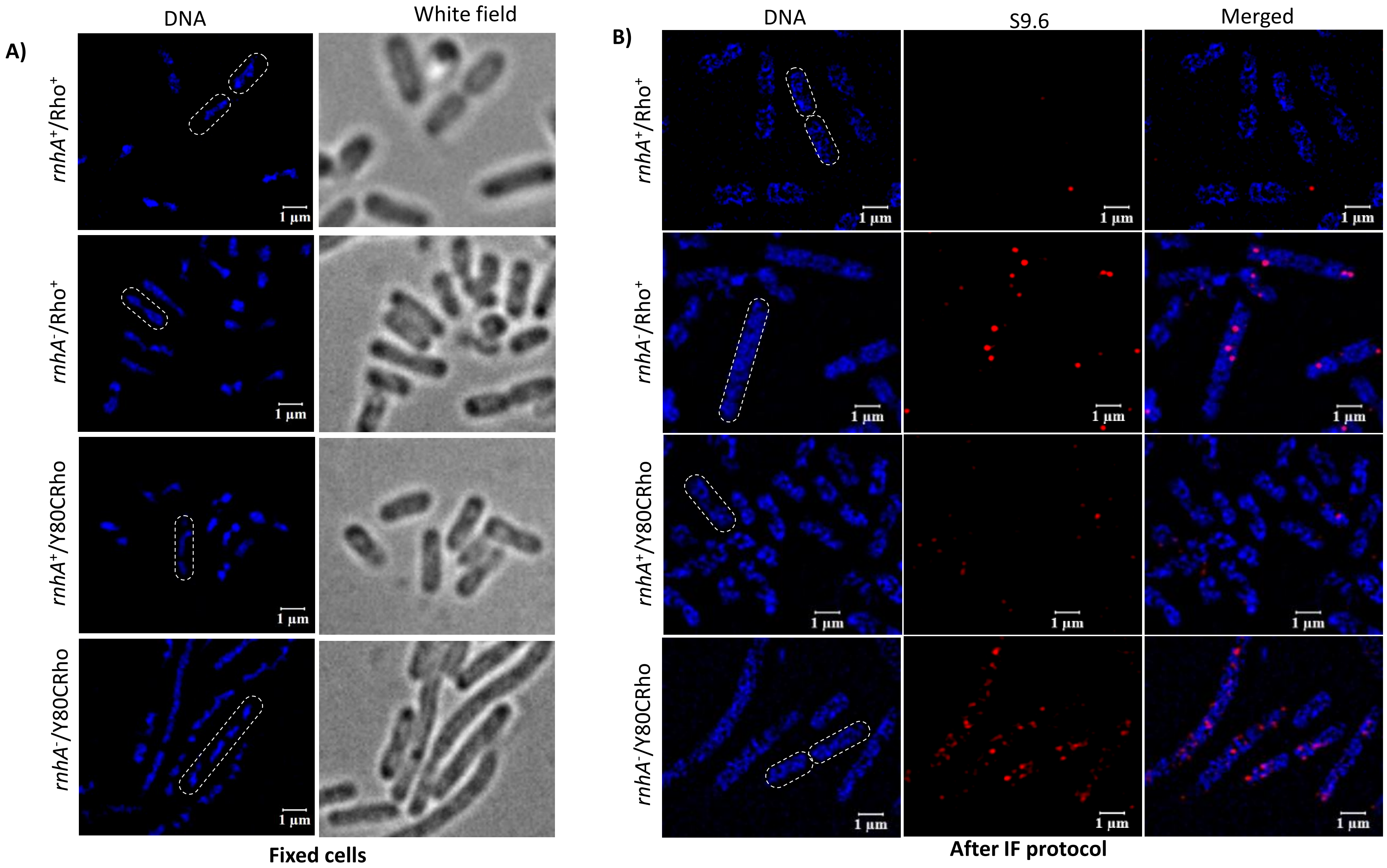

Figure S3 *Morphology of E.coli MC4100 after various treatments for microscopy.* **A)** Super-resolution microscopy images of the paraformaldehyde-fixed *E. coli* MC4100 cells with the genotypes, *rnhA*$^-$/Rho$^+$, *rnhA*$^+$/Y80C Rho, and *rnhA*$^-$/Y80C Rho. Each panel shows cells stained with DAPI (left) and the corresponding white field images (right). m**B)** Super-resolution microscopy images of the *E. coli* MC4100 cells with genotypes, *rnhA*$^-$/Rho$^+$, *rnhA*$^+$/Y80C Rho, and *rnhA*$^-$/Y80C Rho captured after treatments followed in the immunofluorescence microscopy protocol. The images were obtained from the cells probed with mS9.6 antibody (Red), and DNA stained with DAPI (Blue). The imaginary cell contour is drawn with a white dashed line based on the actual size of the cells. The scale bar indicated in all the panels is of 1 µm size.
