# Supplementary material for: The bacterial transcription terminator, Rho, functions as an RNA:DNA hybrid (RDH) helicase in vivo
Source: Biochem J. 2025 May 26;482(11):655–74. doi: 10.1042/BCJ20253089 (PMC12203952; doi:10.1042/BCJ20253089)
Supplement: Online supplementary figure S4 [file BCJ-482-11-BCJ20253089-s005.pdf]

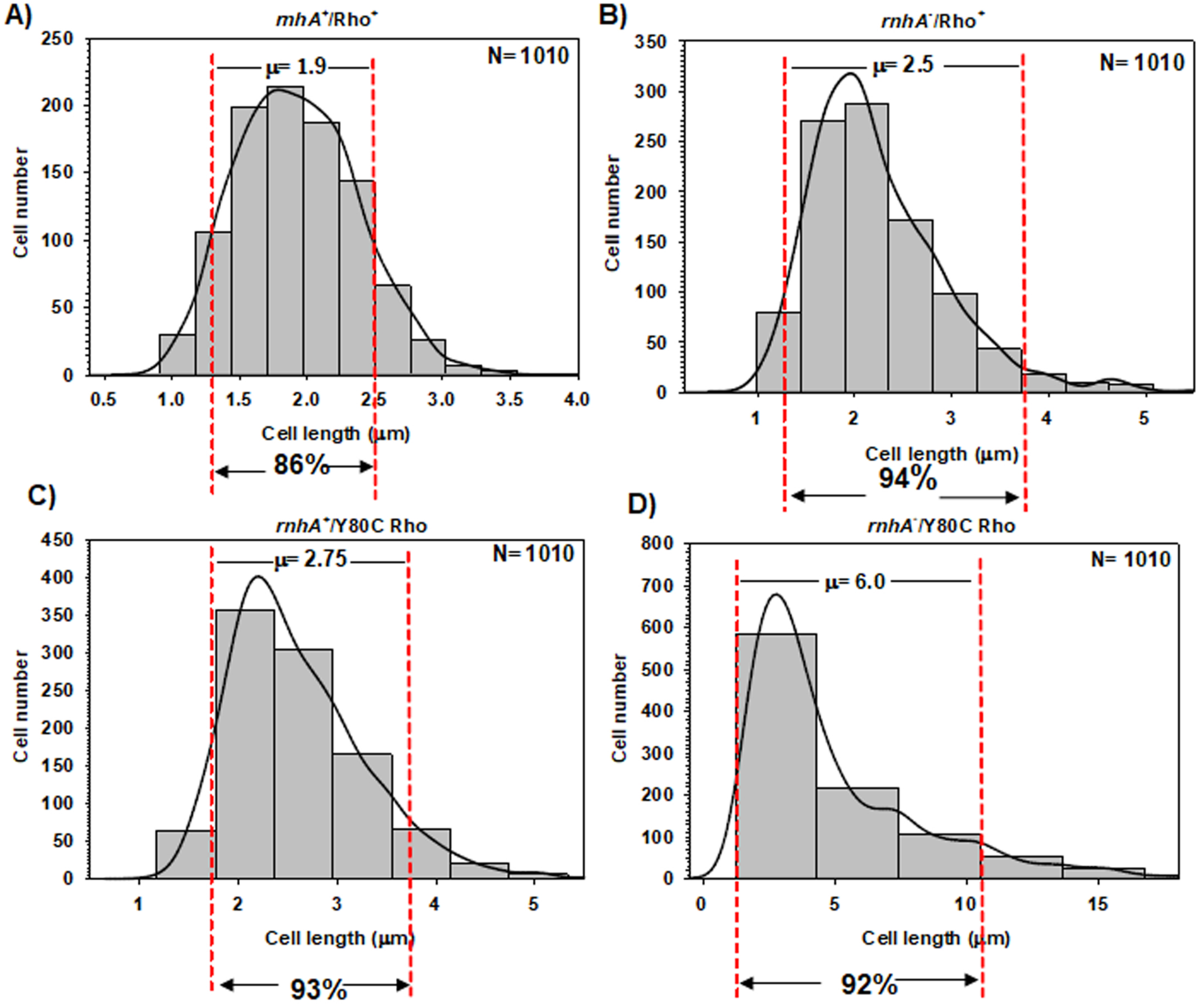

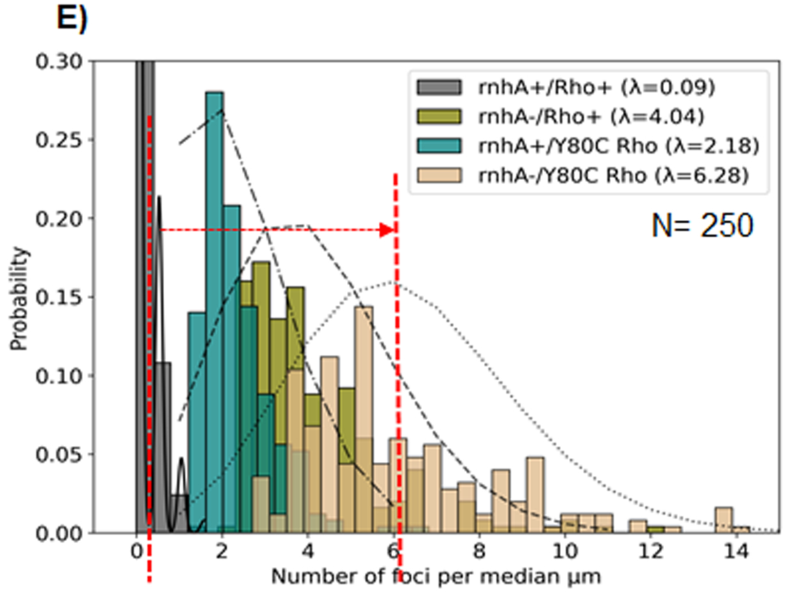

Figure S4. Median cell length calculation. The Gaussian distribution plots showing the distribution of cell length for **A)** *rnhA*+/Rho+ **B)** *rnhA*-/Rho+ **C)** *rnhA*+/Y80C Rho **D)** *rnhA*-/Y80C Rho. The plots were generated by Sigma Plot 15 software with kernel density overlay analysis. μ represents the mean of the distribution and an estimate of the median size of the cell. N is the total number of cells used for distribution analyses. % values represent the percentage of cell numbers in that range of cell length considered for calculation of the μ ignoring the outliers.

E) The Poisson distribution plots show the number of foci per cell per median μm in the case of *rnhA*+/Rho+ (Black), *rnhA*-/Rho+ (Grey) *rnhA*+/Y80C Rho (Orange) and *rnhA*-/Y80C Rho (Olive). The plot was generated by a dedicated Python-inbuilt script. Poisson distribution rate 'λ' represented the average of the foci per cell. 'N' is the total number of cells used for distribution analyses. The red dashed line represents the average value in the case of *rnhA*+/Rho+ (left) and *rnhA*+/Y80C Rho (right).
