# Supplementary material for: The bacterial transcription terminator, Rho, functions as an RNA:DNA hybrid (RDH) helicase in vivo
Source: Biochem J. 2025 May 26;482(11):655–74. doi: 10.1042/BCJ20253089 (PMC12203952; doi:10.1042/BCJ20253089)
Supplement: Online supplementary figure S5 [file BCJ-482-11-BCJ20253089-s006.pdf]

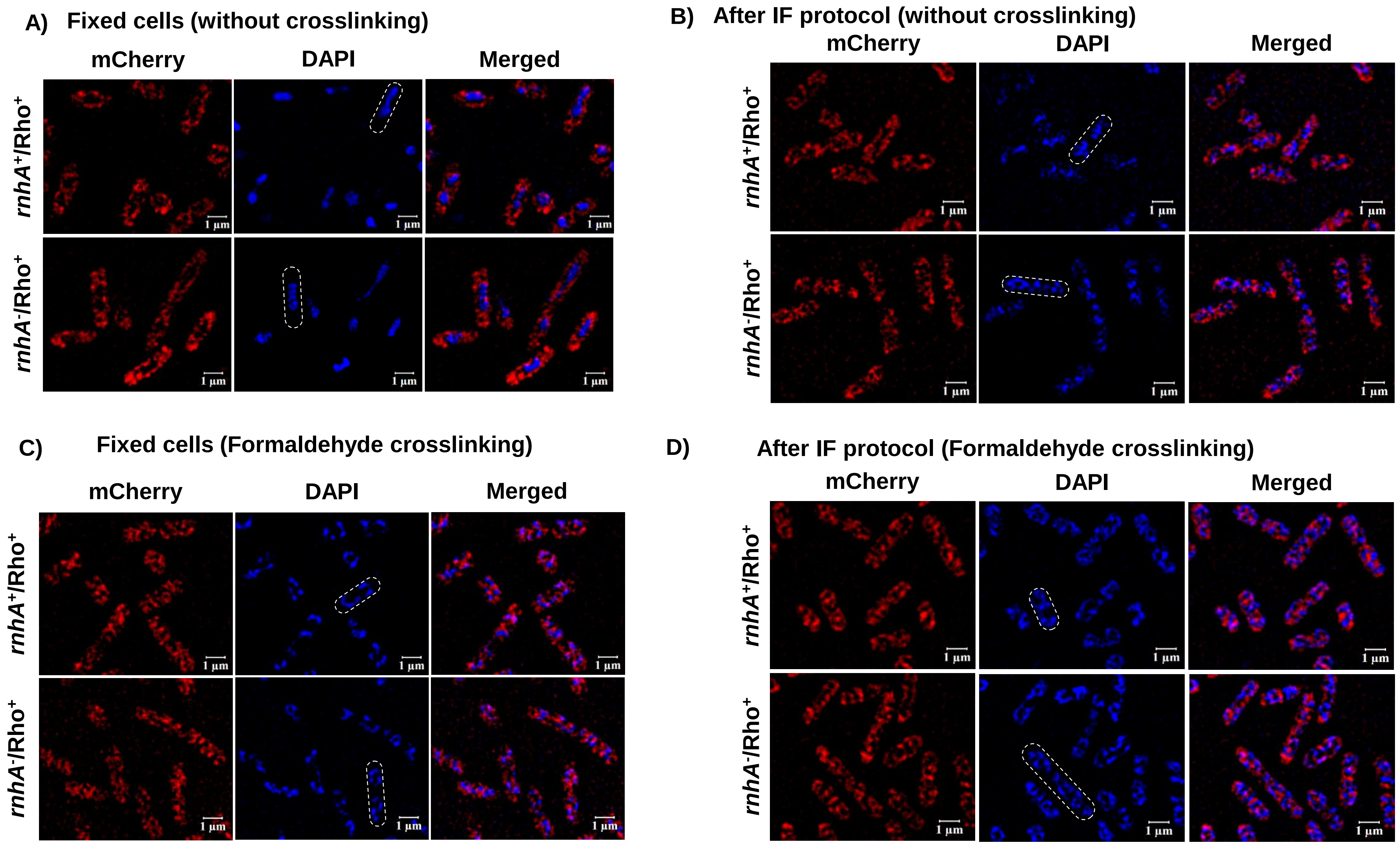

Figure S5. *Changes in DNA organization and Rho localization due to formaldehyde crosslinking and Immunofluorescence (IF) protocols.* Super-resolution microscopy images of the *E. coli* MC4100 *rnhA*$^-$/Rho$^+$ or *rnhA*$^+$/Rho$^+$ cells expressing mCherry Rho. A) Paraformaldehyde-fixed cells without formaldehyde crosslinking, B) cells after IF protocol without formaldehyde crosslinking, C) fixed cells with formaldehyde crosslinking, and D) cells after IF protocol with formaldehyde crosslinking. The images were captured from the cells expressing mCherry-Rho (Red) and DNA stained with DAPI (Blue). The imaginary cell contour is drawn with a white dashed line based on the actual size of the cells. The scale bar indicated in all the panels is of 1 µm size.
