# Supplementary material for: The bacterial transcription terminator, Rho, functions as an RNA:DNA hybrid (RDH) helicase in vivo
Source: Biochem J. 2025 May 26;482(11):655–74. doi: 10.1042/BCJ20253089 (PMC12203952; doi:10.1042/BCJ20253089)
Supplement: Online supplementary figure S6 [file BCJ-482-11-BCJ20253089-s007.pdf]

A) *rnhA*⁺/Rho⁺ B) *rnhA*⁻/Rho⁺

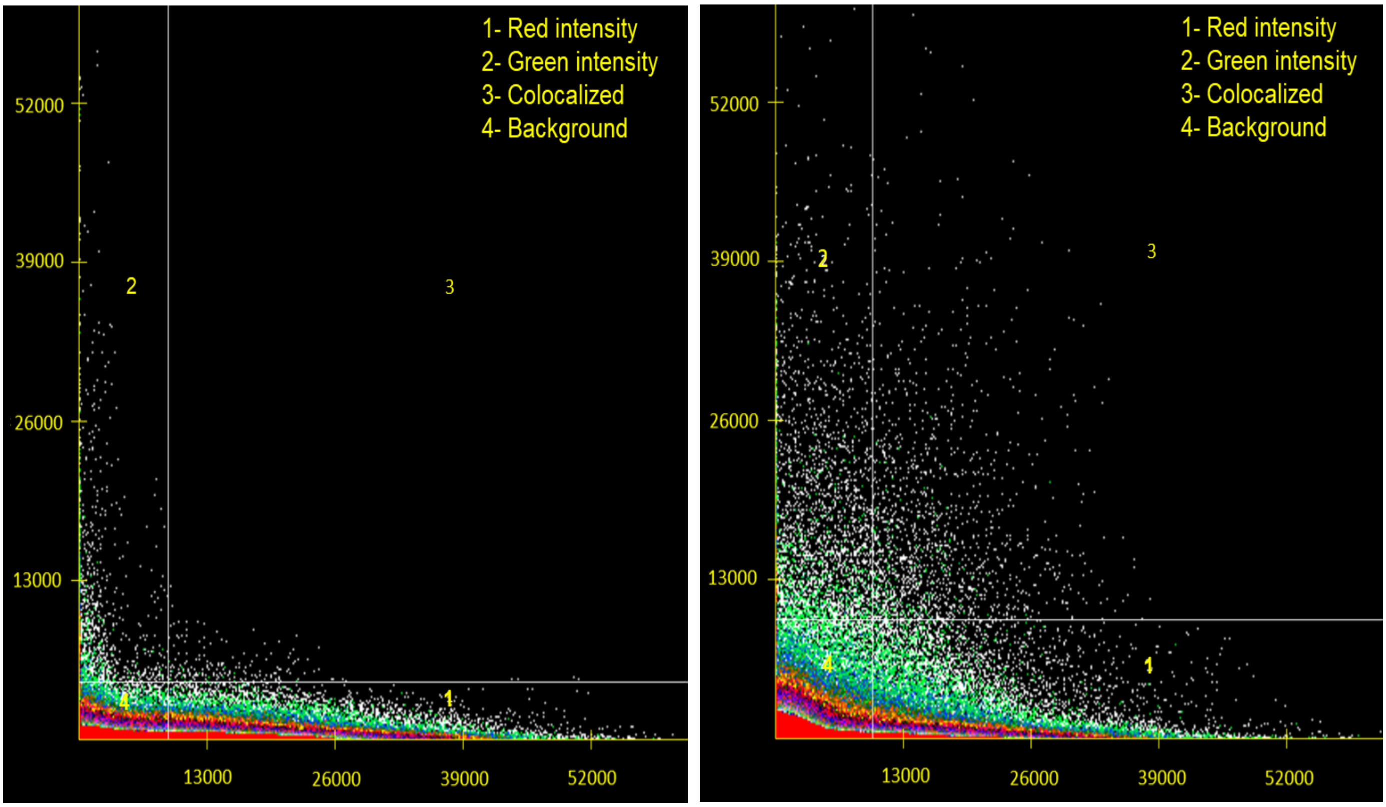

Figure S6: The scattered plot represents the distribution of red and green intensity signals in the strains **(A)** *rnhA*$^{+}$/Rho^{+} and **(B)** *rnhA*$^{-}$/Rho^{+}. The 'X '-axis represents the intensity of the red signal and the 'Y '-axis represents the intensity of the green signal. The 1,2,3, and 4 quadrants represent the red, green, colocalized, and background (noise) signal areas, respectively.
