# Supplementary material for: The bacterial transcription terminator, Rho, functions as an RNA:DNA hybrid (RDH) helicase in vivo
Source: Biochem J. 2025 May 26;482(11):655–74. doi: 10.1042/BCJ20253089 (PMC12203952; doi:10.1042/BCJ20253089)
Supplement: Online supplementary figure S7 [file BCJ-482-11-BCJ20253089-s008.pdf]

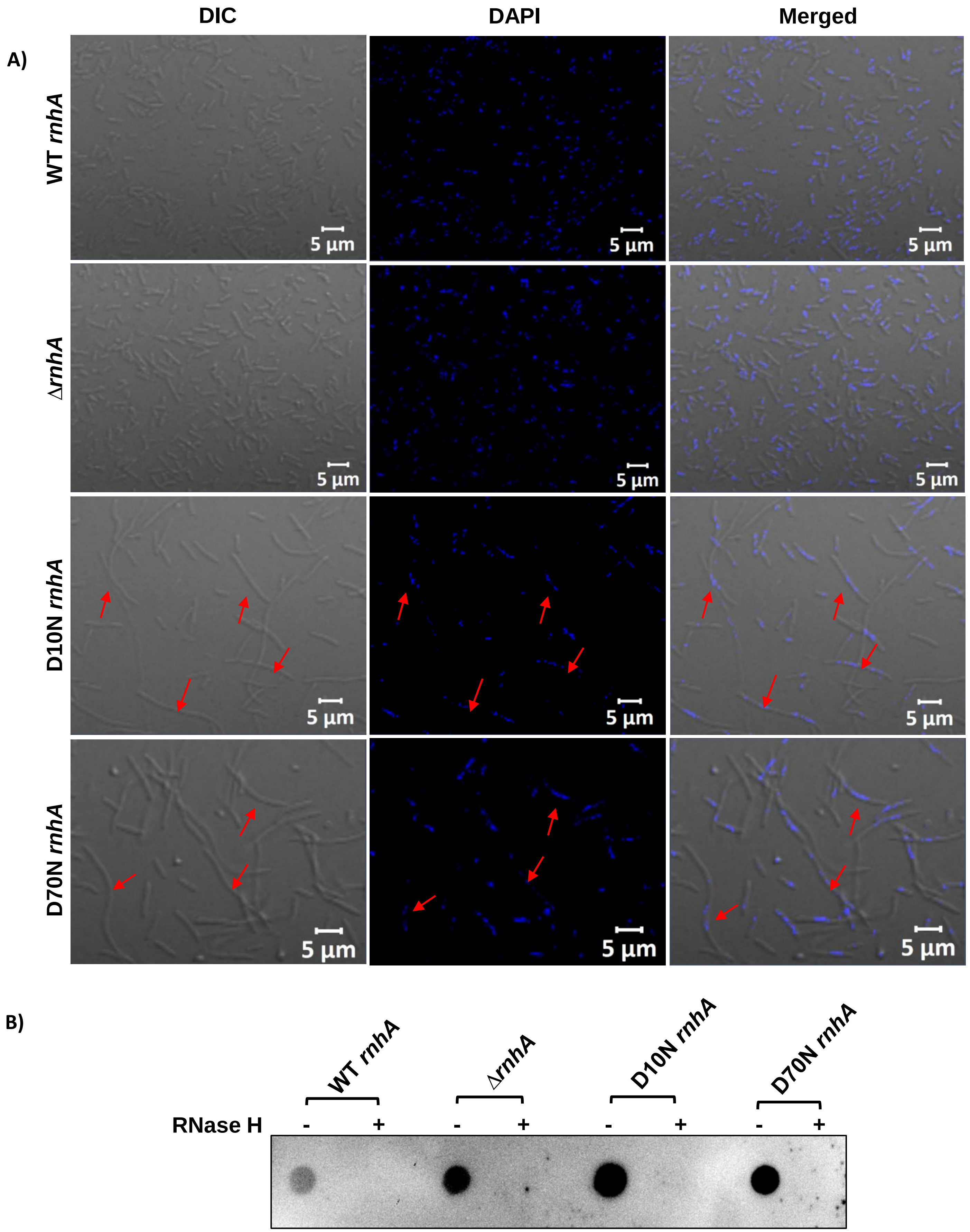

Figure S7: *Change in the cell morphology due to rnhA point mutants*. A) Phase contrast microscopy of the *E. coli* MC4100 cells with the genotypes WT *rnhA*, Δ*rnhA*, and D10N *rnhA or* D70N *rnhA*. The images were obtained by DAPI staining or under the phase-contrast mode (DIC). Merged images of DAPI and DIC in each case are shown. Red arrows indicate cells with unusual shapes. The scale bar indicated as white lines is 5 µm. B) Immunoblotting of the cell extract of the indicated strains spotted on the membranes and were probed with mS9.6 antibody. Other conditions are the same as described in the Figure 3A.
