# Supplementary material for: The bacterial transcription terminator, Rho, functions as an RNA:DNA hybrid (RDH) helicase in vivo
Source: Biochem J. 2025 May 26;482(11):655–74. doi: 10.1042/BCJ20253089 (PMC12203952; doi:10.1042/BCJ20253089)
Supplement: Online supplementary figure S8 [file BCJ-482-11-BCJ20253089-s009.pdf]

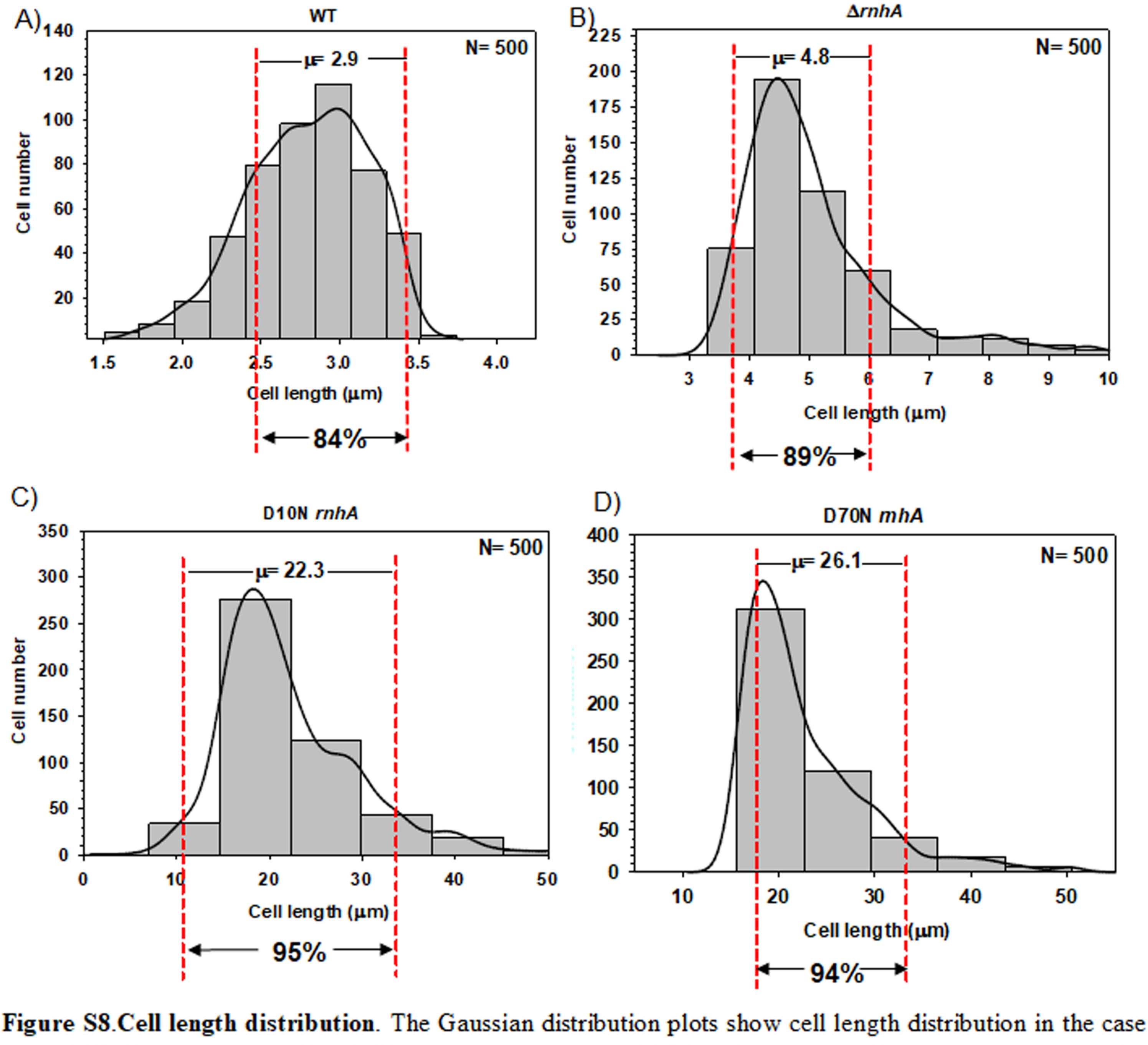

Figure S8.Cell length distribution. The Gaussian distribution plots show cell length distribution in the case of **A)** WT B) Δ*rnhA* strain C) D10N *rnhA* mutant and D) D70N *rnhA* mutant. The plot was generated by Sigma Plot 15 software histogram with kernel density overlay analysis. μ represents the mean of the distribution and gives the estimate of the median lengths of the cells. 'N' is the number of cells used for distribution analyses. % values represent the percentage of cell number present in that cell length range after ignoring the outliers.
