# Supplementary material for: The bacterial transcription terminator, Rho, functions as an RNA:DNA hybrid (RDH) helicase in vivo
Source: Biochem J. 2025 May 26;482(11):655–74. doi: 10.1042/BCJ20253089 (PMC12203952; doi:10.1042/BCJ20253089)
Supplement: Online supplementary figure S9 [file BCJ-482-11-BCJ20253089-s010.pdf]

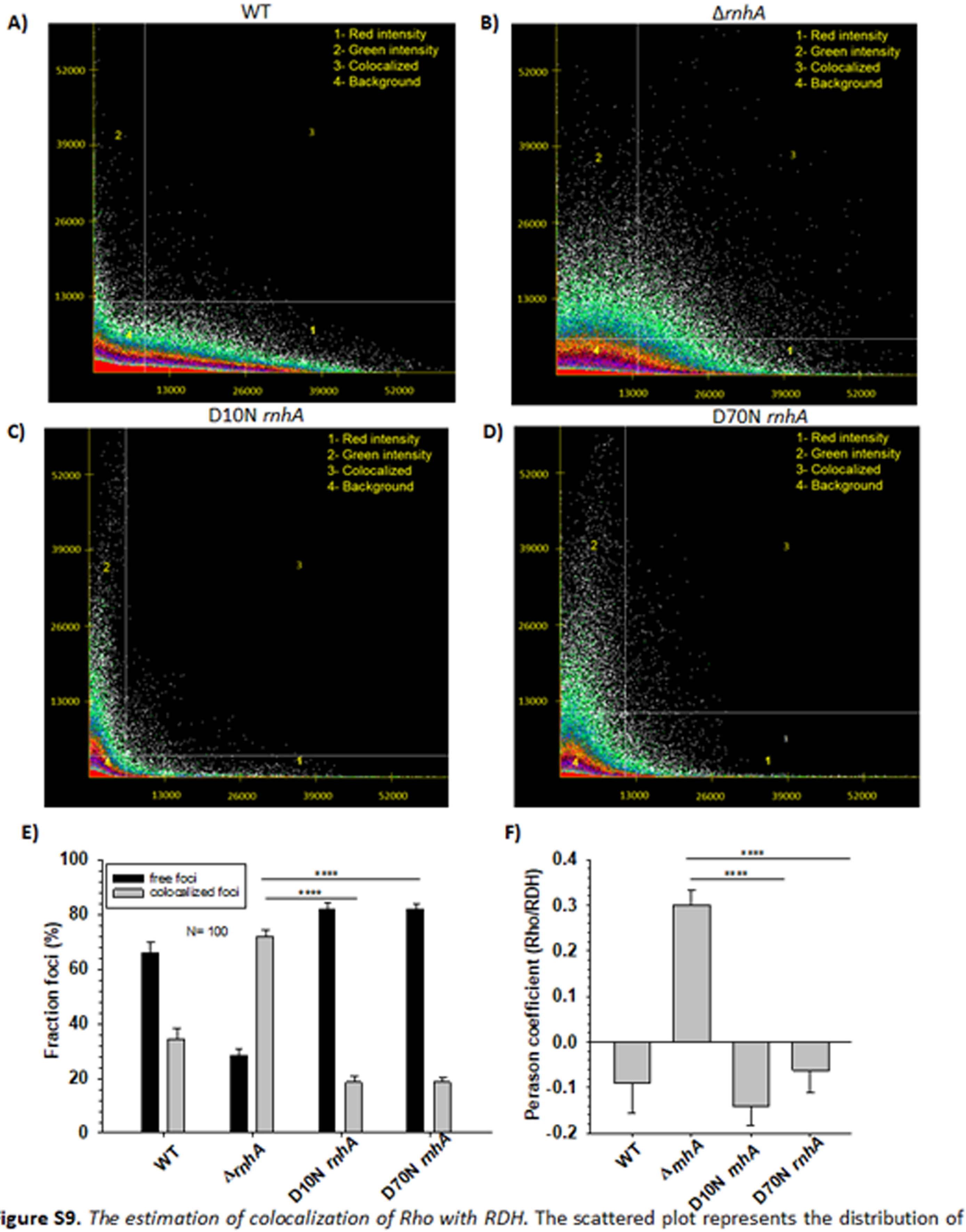

Figure S9. *The estimation of colocalization of Rho with RDH.* The scattered plot represents the distribution of red and green intensity signals in the strains (**A**) WT (**B**) Δ*rnhA* (**C**) D10N *rnhA* and (**D**) D70N *rnhA*. The 'X'-axis represents the intensity of the red signal and the 'Y'-axis represents the intensity of the green signal. The 1,2,3, and 4 quadrants represent the red, green, colocalized, and background (noise) signal areas, respectively. (**E**) The grouped column graph shows the fraction of colocalization of mCherry Rho and RDH spots in the indicated strains. N represents the number of cells used for analyses. (**F**) Plots of the Pearson correlation coefficients of the mCherry Rho and RDH spots in the indicated strains were calculated from (A-D) using Zen software. Error bars represent the SEM. 'N' represents the number of cells used for analyses.
