# Supplementary material for: The bacterial transcription terminator, Rho, functions as an RNA:DNA hybrid (RDH) helicase in vivo
Source: Biochem J. 2025 May 26;482(11):655–74. doi: 10.1042/BCJ20253089 (PMC12203952; doi:10.1042/BCJ20253089)
Supplement: Online supplementary figure S10 [file BCJ-482-11-BCJ20253089-s011.pdf]

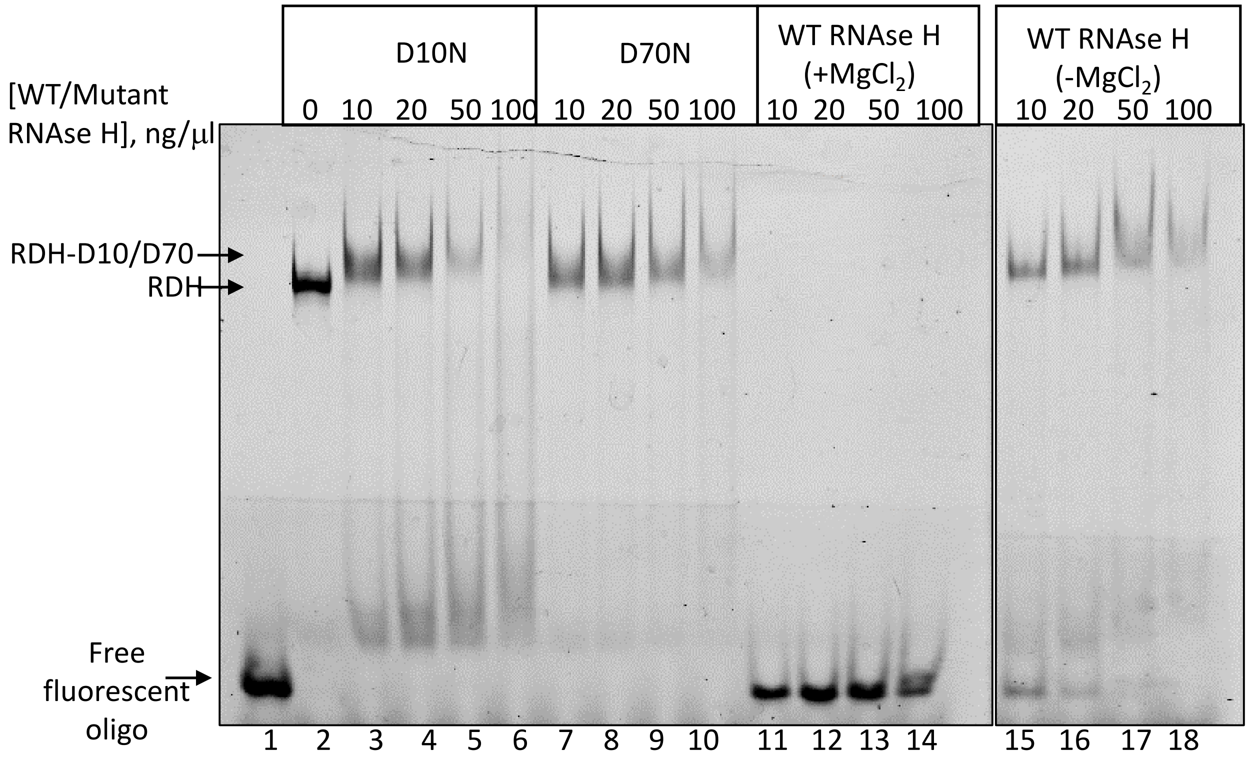

Figure S10. *EMSA assays show the digesting or binding of the RNA-DNA hybrid by WT and RNase H mutants***.** As indicated, increasing concentrations of WT or mutant RNase H were added to the RDH. *In vitro, the* synthesized *λtR1* RNA template was mixed with the 25 nt fluorescent antisense oligos to form the RDH structure at its 3' end as shown in Figure 7A. Free and bound fluorescent oligos in different lanes are indicated. RNase H buffer as described in the methods section was used for all the binding and the RDH unwinding assays.
